# Oral or intranasal immunization with recombinant *Lactobacillus plantarum* displaying head domain of Swine Influenza A virus hemagglutinin protects mice from H1N1 virus

**DOI:** 10.1186/s12934-022-01911-4

**Published:** 2022-09-09

**Authors:** Yufei Zhang, Li Yang, Jiali Zhang, Kun Huang, Xiaomei Sun, Ying Yang, Ting Wang, Qiang Zhang, Zhong Zou, Meilin Jin

**Affiliations:** 1 State Key Laboratory of Agricultural Microbiology, Huazhong Agricultural University, Wuhan, 430070 People’s Republic of China; 2 College of Veterinary Medicine, Huazhong Agricultural University, Wuhan, 430070 People’s Republic of China

**Keywords:** Swine Influenza A virus, *Lactobacillus plantarum*, Mucosal immunity, Intranasal immunization, Oral immunization

## Abstract

**Background:**

Swine influenza A virus (swIAV) is a major concern for the swine industry owing to its highly contagious nature and acute viral disease. Currently, most commercial swIAV vaccines are traditional inactivated virus vaccines. The *Lactobacillus plantarum*-based vaccine platform is a promising approach for mucosal vaccine development. Oral and intranasal immunisations have the potential to induce a mucosal immune response, which confers protective immunity. The aim of this study was to evaluate the probiotic potential and adhesion ability of three *L. plantarum* strains. Furthermore, a recombinant *L. plantarum* strain expressing the head domain of swIAV antigen HA1 was constructed and evaluated for its ability to prevent swIAV infection.

**Results:**

The three *L. plantarum* strains isolated from healthy pig faecal samples maintained the highest survival rate when incubated at pH 3 and at bile salt concentration of 0.3%. They also showed high adherence to intestinal cells. All three *L. plantarum* strains were monitored in live mice, and no major differences in transit time were observed. Recombinant *L. plantarum* expressed swIAV HA1 protein (pSIP401-HA1-ZN-3) and conferred effective mucosal, cellular and systemic immune responses in the intestine as well as in the upper respiratory airways of mice. In conclusion, the oral and intranasal administration of *L. plantarum* strain pSIP401-HA1-ZN-3 in mice induced mucosal immunity and most importantly, provided protection against lethal influenza virus challenge.

**Conclusion:**

In summary, these findings suggest that the engineered *L. plantarum* strain pSIP401-HA1-ZN-3 can be considered as an alternative approach for developing a novel vaccine during an swine influenza A pandemic.

**Supplementary Information:**

The online version contains supplementary material available at 10.1186/s12934-022-01911-4.

## Introduction

Swine influenza A virus (swIAV) is one of the most dominant respiratory pathogens in swine, which often results in a significant economic burden to the pork industry [1]. Additionally, swIAV poses public health concerns owing to its zoonotic potential [1]. Historically, the influenza A(H1N1) virus has triggered several human influenza pandemics [2–5]. More recently, two studies have reported the reassortment of novel influenza viruses in pigs. IAV surveillance of the pig population from 2011 to 2018 in China revealed a recently emerged genotype 4 reassortant Eurasian avian-like H1N1 virus, which contains internal genes from both the triple-reassortant H3N2 and the 2009 pandemic H1N1 viruses [1, 6].

Vaccination remains the most efficient and cost-effective strategy of protecting human and animal populations against IAV [7, 8]. Most currently marketed influenza vaccines are inactivated influenza A virus (IAV) vaccines administered parenterally. The following three types of IAV vaccines exist: whole inactivated virus (WIV) vaccines consisting of formaldehyde- or β-propiolactone-inactivated whole virion, split virus vaccines, and subunit vaccines [8]. Currently, most commercial swIAV vaccines are traditional WIV vaccines, often with oil-in-water adjuvants [9, 10]. However, these commercial vaccines are not regularly updated and do not protect against the large diversity of swIAVs circulating in swine populations [9]. The limited strains currently available in commercial swine influenza vaccines highlight the urgent need for a universal swine influenza vaccine.

In last decade, different approaches have been investigated; among which live bacterial vaccines have been the focus of attention. Most of the sold probiotic LAB belong to the genera of Lactobacillus that are very attractive for vaccine production and delivery because of their ‘generally recognized as safe’ (GRAS) status, adjuvant properties, mucoadhesive ability, easy genetic manipulation, and the availability of industrial production processes [11–15]. Additionally, as live bacterial vaccines are administered orally or nasally, they have higher acceptance and better safety, but also avoid the risk of contamination due to needles and syringes. Effective mucosal immunogenicity and protection after oral and nasal vaccination with Lactobacillus strains expressing antigens has been achieved against several viral and bacterial pathogens [15–22]. Lactobacillus strains may be considered promising vehicles not only for antigens but also for biologically active compounds, such as immunomodulators, antibodies, enzymes, and peptides [13–15].

The aim of the present study was to evaluate the probiotic properties of *Lactobacillus plantarum* strains isolated and identified from the faecal samples of healthy pigs using in vitro assay methods; among them, one strain showing desirable probiotic properties was selected for further assay of vehicles for swIAV antigen effects in mice. In addition, cell wall-anchored *L. plantarum*-HA1 was delivered via oral and intranasal immunisation, and its immunogenicity was evaluated in a mouse model.

## Materials and methods

### Characterization of *Lactobacillus* isolates from swine intestine

Twenty faecal samples were collected from 10 conventionally raised healthy pigs in Wuhan, China. The animals had no history of gastrointestinal disease and had not been administered antibiotics for at least 2 weeks before sampling. Samples were serially diluted in an isotonic sodium chloride solution (0.85% w/v), streaked on de Man, Rogosa and Sharpe (MRS) plates, and incubated at 37 °C for 48 h under anaerobic conditions. Following this, approximately 10–15 randomly selected colonies were plated on MRS agar, to identify the pure culture by colony morphology. All isolates were identified based on their morphological and staining characteristics (gram-positive bacilli) and negative catalase reaction (3% v/v H_2_O_2_). The pure colonies were stored in a stock medium (10% skimmed milk and 20% glycerol) at − 80 °C for further testing.

Identification of the tested isolates was performed using 16S rDNA sequence analysis. Strain DNA was extracted from the bacterial colonies using a QIAamp DNA Mini Kit (Qiagen, Wuhan, China). The 16S rRNA gene was amplified using universal primers 27F (5′-AGAGTTTGATCCTGGCTCAG-3′) and 1492R (5′-GGTTACCTTGTTACGACTT-3′). Purified polymerase chain reaction (PCR) products (one product per isolate) were sent to Sangon Biotech (Shanghai, China) for sequencing. Species were identified by comparing the 16S sequence with bacterial sequences in Medline databases; a score higher than 99% was considered significant. Phylogenetic trees were reconstructed using neighbour-joining and maximum likelihood methods in the MEGA7 program, with bootstrap values based on 1000 replications.

### Evaluation of probiotic properties

To determine the probiotic potential, the following properties were evaluated: tolerance to low pH and bile salts, antibiotic susceptibility, and antimicrobial activity.

#### Resistance to low pH

Bacterial culture (1 mL) was inoculated into five tubes, each containing 9 mL MRS broth adjusted to pH ranging from 2–6. After 16 h of incubation at 37 ℃, the bacterial cells were harvested by centrifugation at 10,000×*g*, 4 ℃ for 5 min and washed twice with phosphate-buffered saline (PBS) (pH 7.2). Resistance to low pH was assessed in terms of viable colony counts and enumerated on MRS agar plates after incubation at 37 ℃.

#### Bile salt tolerance

Tolerance to bile salts was tested as previously described [23]. MRS broth was supplemented with different concentrations (0, 0.1, 0.2, and 0.3%) of bile (Oxgall). These were inoculated with lactobacilli to investigate bile salt tolerance. Bacterial cells from overnight (16 h) cultures were harvested (10,000×*g*, 5 min, 4 ℃) and washed twice with PBS (pH 7.2). Resistance was assessed in terms of viable colony count and enumerated after incubation at 37 ℃. All the values were obtained from three independent experiments.

#### Antibiotic susceptibility test

The agar well diffusion method was used to test antibiotic susceptibility to frequently used antibiotics. The antibiotics evaluated in this study against all ten isolates with the same concentrations are ampicillin (2 mg/L), gentamycin (16 mg/L), erythromycin (1 mg/L), kanamycin (64 mg/L), and clindamycin (4 mg/L). The antibiotic stock solutions were prepared in distilled water according to guidelines from the Clinical and Laboratory Standards Institute and the European Food Safety Authority. Distilled water was used as the control.

#### Antibacterial activity

The antimicrobial activity of the isolated strains was measured using a previously described method, with some modifications [24]. Twenty pathogen strains preserved in our laboratory were used in antimicrobial assays to evaluate the antimicrobial activity of the isolated *L. plantarum* strains. The pathogenic strains included *Escherichia coli ATCC35150*, *Escherichia coli XT-13*, *Escherichia coli AV006*, *Escherichia coli GS-1*, *Salmonella ST*, *Salmonella SH*, *Salmonella SS*, *Salmonella SE*, *Salmonella SO*, *Salmonella pullorum SA023*, *Salmonella enteritidis SA083*, *Salmonella typhimurium SA014*, *Riemerella anatipestifer 2,020,008*, *Riemerella anatipestifer 2,020,014*, *Pasteurella multocida 2,018,133*, *Clostridium perfringens 22*, *Staphylococcus* sp *1*, *Staphylococcus* sp *2*, *Staphylococcus aureus*, and *Staphylococcus epidermidis*. Each pathogen was coated on a Luria–Bertani (LB) agar plate. Following this, the Oxford cup was carefully placed in the plate and 200 μL of overnight *L. plantarum* culture was placed inside the Oxford cup. The plate was incubated at 37 °C for 24 h, and the inhibition zone diameters were measured.

### Bacterial strains and growth conditions

The bacterial strains and plasmids used in the present study are listed in Table 1. *L. plantarum* strains were cultured in MRS broth at 37 ℃ without shaking. *Escherichia coli* DH5ɑ used in the transformation experiments involving the subcloning of DNA fragments was cultivated in LB broth at 37 ℃ with agitation. When appropriate, erythromycin was used at a concentration of 10 and 200 µg/mL for *L. plantarum* strains and *Escherichia coli*, respectively, both in broth and solid media.

**Table 1:** Strains, plasmids, and primers used in this study

Strain, plasmid, or primer	Relevant characteristic	Source or reference
Strain		
* E. coli* DH5ɑ	Cloning host	TransGen Biotech
* L. Plantarum* MQDR2	Cloning host	This study
* L. Plantarum* A37	Cloning host	This study
* L. Plantarum* M3	Cloning host	This study
* L. Plantarum* 185,362	Cloning host	This study
* L. Plantarum* ZN-3	Cloning host	This study
Plasmid		
pSIP401	*spp*-based expression vector, Erm^r^	
pSIP401-*mcherry*	pSIP401 derivative, *mcherry* controlled by P*sppA*, Erm^r^	This study
PUC57-IRFP713	Gene synthesis	This study
pSIP401-IRFP713	pSIP401 derivative, IRFP713 controlled by P*sppA*, Erm^r^	This study
PUC57-HA1	Gene synthesis	This study
PUC57-SP1216-2578	Gene synthesis	This study
pSIP401-SP1216-2578	pSIP401 derivative,CWA200 fused to the spLp_2588; Erm^r^	This study
pSIP401-SP1216-HA1-2578	pSIP401 derivative, HA1-CWA200 controlled by P*sppA*, Erm^r^	This study
Primer		
401-mcherry-F	CCGCCATGGATGGTATCAAAAGGAGAAGAAGA	Nco I
401-mcherry-R	CCCAAGCTTTTATTTATATAATTCATCCATACCA	Hind III
401-IRFP713-F	CCGCCATGGATGGTGTATACGGAAAATACGGGTAAGCA	Nco I
401-IRFP713-R	CCCAAGCTTTTATTCTTTATGTTGCCGTTTTGCC	Hind III
HA1-F	CGCGGTACCTCCCTTAATGGTAAAATTCCA	Kpn I
HA1-R	GCCGGATCCATGAACTTGAGCATCTGA	BamH I
PUC57-SP1216-HA1-2578-F	taggagtatgattcccatggTGAAGAAGTTTAATTTTAAGACGAT	Seamless Assembly
PUC57-SP1216-HA1-2578-R	cgtgctgtaatttgaagcttTTAAGCACGACGCCGATAACCA	Seamless Assembly
401-SP1216-HA1-2578-F	taggagtatgattcccatggTGAAGAAGTTTAATTTTAAGACGAT	Seamless Assembly
401-SP1216-HA1-2578-R	cgtgctgtaatttgaagcttTTAAGCACGACGCCGATAACCA	Seamless Assembly
NP-F	TGGAGGGGTGAAAATGGACG	QPCR
NP-R	CCTGGGTTGCGACTTTCTCT	QPCR

### DNA manipulations and plasmid construction

Primers used in this study are listed in Table 1. All plasmids used in this study for expression in *Lactobacillus* spp. are derivatives of the modular pSIP401 vector series, constructed and developed for inducible gene expression, secretion, and surface anchoring of proteins [25–29]. pSIP401-mcherry was constructed by amplifying the mCherry protein from pMcherry-C1 using the primer pair 401-mcherry-F/401-mcherry-R (Table 1), following which the resulting 0.7 kb PCR fragment was digested with Nco I/Hind III. The IRFP713 gene fragment was optimised for expression in *L. plantarum*, synthesised using GenScript (Nanjing, China), and cloned into a pSIP401 plasmid, yielding pSIP401-IRFP713.

The HA1(H1N1) and SP1261-2578 gene fragments were optimised for expression in *L. plantarum,* synthesised using GenScript (Nanjing, China), and cloned into a pUC57 plasmid, yielding pUC57-HA1 and pUC57-SP1216-2578, respectively. These plasmids contain N-terminal signal peptides (SP) derived from genes encoding Lp_1261 [30]. The total length of the Lp_1261 anchor is 75 residues, including 22 amino acids in the SP. The cell wall anchor sequence comprises 223 C-terminal residues from Lp_2578, of which 189 residues are the linker region, followed by the LPQTSE motif, which is followed by a hydrophobic stretch and a positively charged C-terminal [25, 30–32]. The plasmid for the intracellular production of SP1216-HA1-2578 was constructed by amplifying the HA1 fragment using the primer pair PUC57-SP1216-HA1-2578-F and PUC57-SP1216-HA1-2578-R (Table 1), using pUC57-HA1 as a template. The resulting PCR fragment was ligated into the KpnI/BamHI-digested vector PUC57-SP1216-2578 using an In-Fusion® HD Cloning Kit (Clontech, Dalian, China) to yield the plasmid PUC57-SP1216-HA1-2578. pSIP401-SP1216-HA1-2578 was constructed by amplifying the SP1216-HA1-2578 open reading frame from PUC57-SP1216-HA1-2578 using the primer pair 401-SP1216-HA1-2578-F/401-SP1216-HA1-2578-R (Table 1), following which the PCR fragment was ligated into the NcoI/HindIII-digested vector, pSIP401, using the In-Fusion^®^ HD Cloning Kit.

### Gene expression in *Lactobacillus plantarum*

To generate the four expression strains, pSIP401-mcherry, pSIP401-IRFP713, and pSIP401-SP1216-HA1-2578 were transformed into electro-competent *L. plantarum* strains and transformants were selected on MRS agar plates containing 5 μg/mL erythromycin. Gene expression was performed by diluting the overnight cultures of *L. plantarum* strains harbouring the plasmids in 100 mL of fresh pre-warm MRS broth (for erm-based systems, 5 µg/mL of erythromycin was added) at an OD_600_ of approximately 0.1, and incubated at 37 ℃ without agitation. The cells were induced at OD_600_ of 0.3 by adding the peptide pheromone IP-673 [33] to a final concentration of 25 ng/mL. The cultures were harvested 6 h after induction by centrifugation at 5,000×*g* for 5–10 min. The cells were washed once in PBS and stored at − 20 ℃, before staining or sodium dodecyl sulphate–polyacrylamide gel electrophoresis (SDS-PAGE) analysis.

### Flow cytometry and indirect immunofluorescence microscopy

Bacterial cultures were grown and induced as previously described [34]. Cells from approximately 0.5 mL of culture were harvested once with PBS, washed four times with PBS, and resuspended in 1 mL PBS without bovine serum albumin (BSA) at 4 °C. The resulting bacterial suspensions were immediately analysed by flow cytometry using a MACSQuant analyser (Miltenyi Biotec GmbH, Bergisch Gladbach, Germany), according to the manufacturer's instructions.

For indirect immunofluorescence microscopy, the stained cells were resuspended in 100 µL of PBS and stained with monoclonal anti-c-Myc antibody and goat anti-mouse Alexa Fluor^®^ 594 (IgG H&L) (Abcam; Cambridge, UK) and visualised under an Axio Observer.z1 microscope (Zeiss, Oberkochen, Germany) using excitation and emission wavelengths of 591 and 614 nm, respectively, and a bright field photomultiplier tube for transmitted light.

### Assay of adhesion to Caco-2 cells

Caco-2 cells were routinely grown in Dulbecco’s modified Eagle medium (DMEM) supplemented with 10% foetal bovine serum, 100 IU/mL penicillin G, and 100 μg/mL streptomycin. For adhesion assays, 10^5^ Caco-2 cells were seeded in 24-well plates with glass cover slips and maintained at 37 °C under 5% CO_2_ for 3 days. Prior to the experiments, all bacterial cultures were harvested until the stationary phase or after induction, washed twice in PBS, and suspended in DMEM without antibiotics at 1.5 × 10^8^ colony forming units (CFU)/mL. Cells were co-incubated for 2 h (5% CO_2_ atmosphere at 37 °C). After co-incubation, non-adherent bacteria were washed twice with PBS. For microscopy, Caco-2 cells were seeded onto a glass coverslip and cultivated for 24 h. Subsequently, the cells were fixed with 4% paraformaldehyde and stained with 0.1% crystal violet staining solution. The experiments were performed in duplicates. The percentage of Caco-2 cells (1,000 cells) adhering to at least one *Lactobacillus* species was counted.

### Transmission electron microscopy

The bacteria were resuspended in fresh DMEM and added to the prepared Caco-2 monolayers to be cultured in 6-well plates at 37 °C with 5% CO_2_ for 2 h. The monolayers were then washed five times with PBS to remove unbound bacteria. The samples were then fixed with glutaraldehyde solution for 6 h. Caco-2 cells were stained with phosphotungstic acid and observed using H-7650 transmission electron microscopy (Hitachi, Tokyo, Japan).

### In vivo fluorescence imaging

Fluorescence imaging was performed using a multimodal IVIS Lumina XR imaging system (PerkinElmer). The mice were euthanised by cervical dislocation, followed by exposure of the abdominal cavity and removal of the intestine from the stomach to the rectum [33]. The instrument background fluorescence was removed using an adaptive fluorescence background subtraction tool. Images were analysed using Living Image version 4.3.1 [33].

### Colony detection of strains in mice intestines

**Fig. 1:**
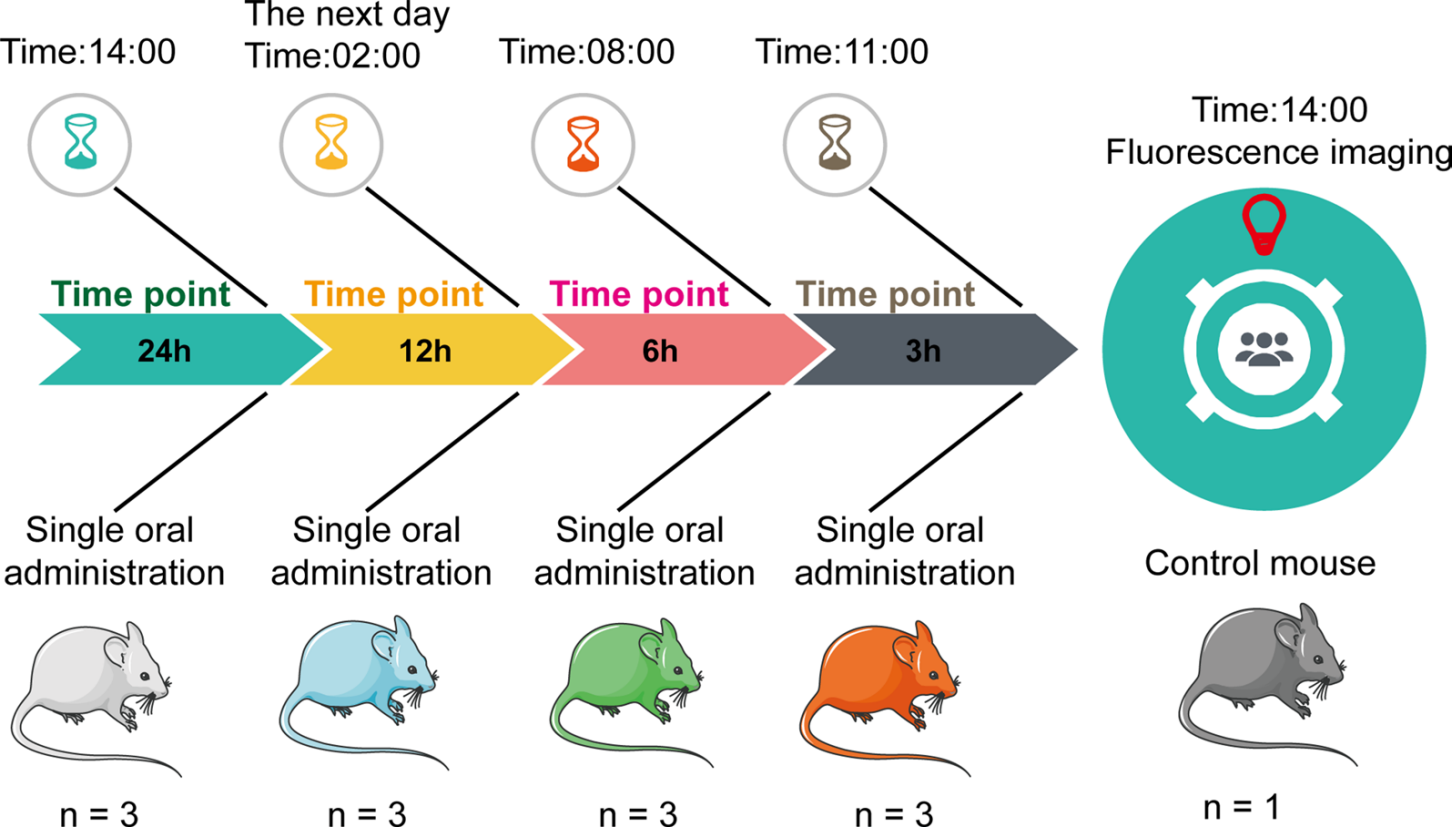
Experimental scheme for the in vivo imaging of fluorescent recombinant L. plantarum within the digestive tract of mice

A total of 48 BALB/c female 6-week-old mice were received 10^9^ CFU/100 µL fluorescent recombinant L. plantarum. The intestines were harvested immediately after the animals were euthanized at eight time points (2, 4, 6, 12, 24, 48, 72, and 96 h; n = 6 mice in each group). At each time point, the gastrointestinal tract (GIT) of mice was collected and imaged using an IVIS Lumina XRMS Series III (PerkinElmer) (Fig. 1); the status of probiotics in the mouse intestine was observed at different time points. Simultaneously, the small intestine (the duodenum and jejunum) and the large intestine (the caecum and colon) of each mouse were segmented, PBS was used to wash the intestinal mucosa and the attached contents, and the abundance of probiotics was quantified using the plate coating method.

### Western blotting

The protein extracts, in amounts that were adjusted based on the OD_600_ at harvesting, were separated by SDS-PAGE using 10% Mini-Protean TGX precast gels (Bio-Rad) and transferred to a nitrocellulose membrane. The monoclonal murine anti-Myc antibody (ABclonal, Wuhan, China) and used as recommended by the manufacturer. Protein bands were visualised using a polyclonal rabbit anti-mouse antibody conjugated with horseradish peroxidase (HRP) (ABclonal, Wuhan, China).

### Oral and intranasal immunization with recombinant *Lactobacillus plantarum*

The wild-type and recombinant *L. plantarum* cultures grown in this study were resuspended to 10^9^ CFU/100 µL for oral immunisation. The immunisation procedure was divided into the following three phases: prime immunisation (days 1, 2, and 3), booster immunisation (days 14, 15, and 16), and the last immunisation (days 29, 30, and 31), with PBS containing 10^9^ CFU *L. plantarum* or 100 µL PBS (negative control) being administered by oral gavage.

Six- to eight-week-old female BALB/c mice (10 per group) received either recombinant bacteria expressing HA1 or the respective control bacteria harbouring the empty vector. An additional control group was administered isotonic sodium chloride solution. *L. plantarum* strains were grown until cultures reached an OD_600_ value of 2.0, and the bacteria were collected by centrifugation (4,000×*g*, 20 min at 20 °C), washed with saline, and suspended at 10^7^ cells in 10 μL. For nasal immunisation, mice were anaesthetised with 2.5% avertin (0.02 mL/g body weight) and 1 μL of an isotonic sodium chloride solution suspension containing 10^7^ cells was inoculated into the nostrils with the help of a micropipette on days 1, 14, and 29.

### Enzyme-linked immunosorbent assay for the detection of HA-specific antibodies

Bronchoalveolar lavage fluid (BALF), intestinal lavage fluid (ILF), and serum samples were collected on day 7 post-immunization, as described previously [35–37]. Briefly, 1 mL sterile PBS was injected into the lungs via the trachea and following three rounds of flashing, the washes were collected and stored at  − 20 °C until analysis. Intestinal and faecal samples were added to 1 mL PBS containing 1% BSA and 1 mM phenylmethylsulfonyl fluoride. After incubation at 4 °C for 12 h, the tubes were vortexed to disrupt all solid material, and then centrifuged at 16,000×*g* for 10 min to collect the supernatants. Blood collected from mice was allowed to clot at 20 °C for 1 h, followed by centrifugation at 1000–2000×*g* for 10 min at 4 °C. Serum was stored at − 20 °C until analysis.

Antigen-specific serum immunoglobulin (Ig)G and faecal secretory IgA (sIgA) antibodies were detected using enzyme-linked immunosorbent assay (ELISA). A square titration in 96-well ELISA microplates (Corning Inc., USA) was implemented to optimize the conditions for detection according to a classical indirect ELISA protocol [38]. ELISA tests were performed on sub-samples from the original 27 mice BALF, ILF, and serum samples at 1, 2 and 3 weeks after immunization, using all conjugate-blocking agent combinations. At any test-time, there 96-well ELISA microplates blocked with the same agent were used.

### Hemagglutination inhibition test

Sera from immunized mice were collected for HI assays carried out as described previously [39]. The sera were pretreated with receptor-destroying enzyme overnight and heated to 56 °C for 30 min. The sera were two-fold serially diluted with PBS, and incubated with 4 HA units in 96-well microtiter plates for 30 min at 37 °C. This was followed by adding equal volumes of fresh 1% (V/V) chicken red blood cells for 30 min at 37 °C. The HAI titer was defined as the reciprocal of the last dilution with no HA activity.

### Neutralization (VN) assays

The virus neutralization (VN) assay was carried out in MDCK cells as previously described procedure [40]. Serum neutralizing antibody titers were determined by seeding MDCK cells at 1.5 × 104 cells/well in 96-well culture plates and culturing at 37 °C in 5% CO2 to form a monolayer. Serial two-fold dilution of sera samples (starting dilution, 1:10) were made in 96-well cell culture plates using DMEM with 0.3% bovine serum albumin (BSA) containing 100 U/mL penicillin and 100 μg/mL streptomycin, and then an equal volume (50 μL) of diluted virus containing 100 TCID50 was fixed to the diluted serums and incubated for 1 h at 37 °C. 100 μL of both serum and virus mixture was transferred to 96-well plates containing a 90% confluent monolayer of MDCK cells and incubated at 37 °C. The cells supernatant was harvested after 48 h and further verified by HA assay.

### Splenocytes proliferation assay

To analyze cell-mediated immune responses, 1 week after the third immunization, the splenocytes were harvested from the spleen of three mice per group. Individual spleens were mechanically disrupted in sterile RPMI-1640 medium (Thermo Fisher Scientific, Shanghai, China) supplemented with 10% heat-inactivated fetal calf serum (FCS, Hyclone, Shanghai, China) and filtered through a 100 µm nylon filter. After incubation with NH_4_Cl lysing buffer (Beyotime Biotechnology, Beijin, China) to remove red blood cells, total spleen cells were adjusted to 5 × 10^6^ viable cells/mL in complete medium consisting of RPMI-1640 medium supplemented with 10% FCS, 2 mM L-glutamine, 50 µM β-mercaptoethanol and 100 U/mL penicillin–streptomycin (Thermo Fisher Scientific, Shanghai, China).

Splenocytes at a density of 2 × 10^5^ cells/well were seeded in each well of a 96 MicroWell plate and stimulated at 37 °C in a humidified atmosphere containing 5% CO_2_ for 48 h with recombinant H1N1 HA1 antigen (10 μg/mL) as the specific antigen for the vaccine groups, concanavalin A (ConA, 10 μg/mL) for the positive control, or without any stimulating antigen. All samples were run in triplicate for each mouse. At 48 h post-stimulation, lymphoproliferation was evaluated by adding 30 μL of 5 mg/mL MTT solution to per well. After further incubation for 4 h, 100 μL of dimethyl sulfoxide was added to each well to dissolve formazan crystals, and the color intensity was measured at 540 nm. The results were reported as a stimulation index using the following calculation [41]:

$${\text{Stimulation index}}\, = \,({\text{Experimental group OD value}}{ - }{\text{blank group OD value}}) \, / \, ({\text{negative control group OD value}}{ - }{\text{blank group OD value}})$$

### Splenocyte stimulation and cytokine analysis

The concentration of Th1 (IFN-γ, IL-12) and Th2 (IL-4) cytokines present in the supernatants of splenocytes from control and test animals were evaluated using commercially available cytokine-specific ELISA kits (R&D, Shanghai, China) as per the manufacturer's instructions. For this, the mononuclear cells were isolated and stimulated as mentioned above. Forty-eight to 72 h post-stimulation, the supernatants were collected and then assayed for the presence of respective cytokines using a quantitative sandwich ELISA assay. Two-fold dilutions of recombinant mouse cytokines were used to generate standard curves. Sample dilutions giving OD450 readings in the linear portion of the appropriate standard curve were used to quantify the levels of each cytokine. For each mouse, all tests were performed in triplicate.

### Viral challenge, clinical observation, and histopathological examination

Mice were anaesthetised intraperitoneally with 2.5% avertin (0.02 mL/g body weight) and then inoculated intranasally with H1N1 (1 × 10^5^ TCID_50_/0.1 mL) and H3N2 (1 × 10^5^ TCID_50_/0.1 mL). To assess the protective efficacy of recombinant *L. plantarum*, all mice were observed for general physical activity and pathophysiological parameters (body weight, fur ruffling, and conjunctivitis).

The animals were euthanised on day 7, and the lungs were collected. The lung tissues of mice in each group were fixed in 10% neutral buffered formalin, embedded using established methods in paraffin, sectioned at 4 mm thickness, and stained with haematoxylin and eosin (H&E). The slides were then observed under a light microscope (Nikon, EX200) to detect histopathological lesions in the lungs. The scoring method was 2 points for inflammatory cell infiltration, 1 point for bleeding, and 1 point for alveolar incompleteness. They were rated blindly by three individuals.

### Statistical analyses

All data in the experiment were obtained from at least three independent experiments and are expressed as the mean ± standard error of mean. Differences were tested using the GraphPad Prism software (version 5.0). Among experimental groups at different time intervals, one-way analysis of variance (ANOVA), with post hoc Bonferroni’s multiple comparison test, was used to determine the statistical significance of cytokine and sIgA levels and two-way ANOVA with post hoc Bonferroni’s multiple comparison test was used to determine significant differences in IgG levels. Survival percentages were analysed using the Kaplan–Meier method. A p-value less than 0.05 was considered statistically significant, and a p-value less than 0.01 was considered highly significant.

### Ethics statement

Specific pathogen-free BALB/c mice were obtained from the Laboratory Animal Services Centre (Huazhong Agricultural University). All animal experiments were conducted in accordance with the recommendations of the Guide for the Care and Use of Laboratory Animals of the Research Ethics Committee, Huazhong Agricultural University, Hubei, China (approval number HZAUMO-2020-0007).

## Results

### Lactobacilli were abundantly present in pig faeces

Overall, 188 strains were isolated from the samples collected in our study. Isolates were identified by 16S RNA amplification. Among these strains, 103 isolates were characterised as lactic acid bacteria, including 80 *Lactobacillus* and 16 Coccus isolates. *Lactobacillus* strains included *Lactobacillus johnsonii* (27 isolates), *Lactobacillus reuteri* (17 isolates), *Lactobacillus zeae* (three isolates), *Lactobacillus rhamnosus* (five isolates), *Lactobacillus casei* (four isolates), *Lactobacillus paracasei* (five isolates), *Lactobacillus amylovorus* (one isolate), *Lactobacillus plantarum* (six isolates), *Lactobacillus harbinensis* (six isolates), *Lactobacillus perolens* (two isolates), *Lactobacillus coryniformis* (two isolates), and *Lactobacillus brevis* (two isolates). The Cocci included *Pediococcus acidilactici* (five isolates), *Pediococcus pentosaceus* (six isolates), and *Enterococcus faecalis* (five isolates).

PCR amplification of the 16S rDNA of *L. plantarum* (six isolates) resulted in a single band of approximately 1500 bp, which corresponds to the expected size of the 16S rDNA gene. Both forward and reverse sequences were obtained and an assembly sequence was generated. The comparative analysis of *L. plantarum* (six isolates) sequences with the published sequences of different *Lactobacillus* species, using the MEGA7 program, demonstrated the phylogenetic distances in a generated neighbour-joining rooted tree (Additional file 1: Fig. S1).

### The amount of overexpression was estimated by comparing the expression level of exogenous proteins

**Fig. 2:**
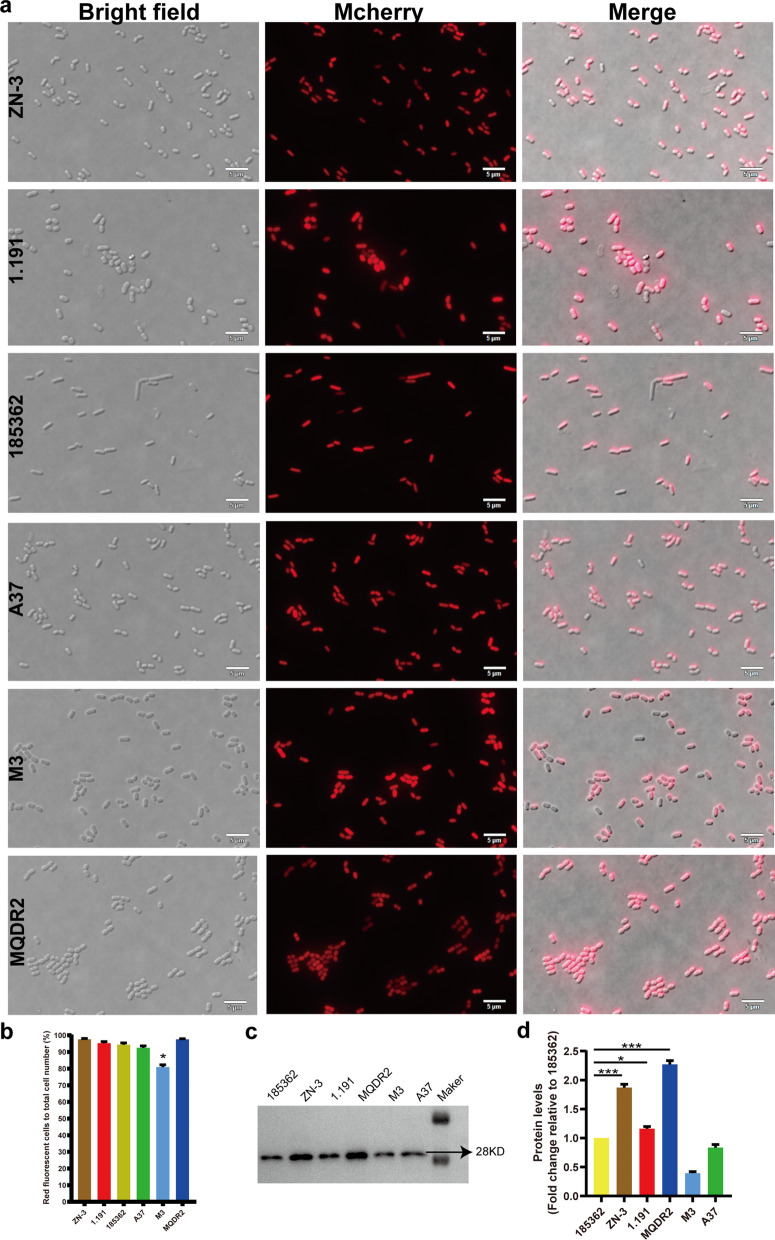
Immunofluorescence microscopy and flow cytometry analysis of expression of mCherry in six strains of *Lactobacillus plantarum*. **a** Observation under fluorescence microscopy. **b** Flow cytometry analysis was used to calculate percentage of total cells that were fluorescent. **c** The Western blots (WB) were probed with an anti-mCherry antibody. **d** The relative gray date from WB experiments (*p < 0.05, **p < 0.01, ***p < 0.001)

To determine the ability of the six *L. plantarum* isolates to express foreign proteins, we constructed recombinant *L. plantarum* expressing mCherry, the expression of which was analysed using fluorescence microscopy, flow cytometry and western blot (Fig. 2). The western blot results showed that the expression of 185,362, A37, m3, and mCherry were low, whereas the expression of ZN-3, 1.191, and MQDR2 mCherry were high (Fig. 2c and d). Thus, we subsequently evaluated the biological characteristics of ZN-3, 1.191, and MQDR2 and screened them for the most suitable mucosal immune vector.

### Resistance to gastric juice and bile salts

**Fig. 3:**
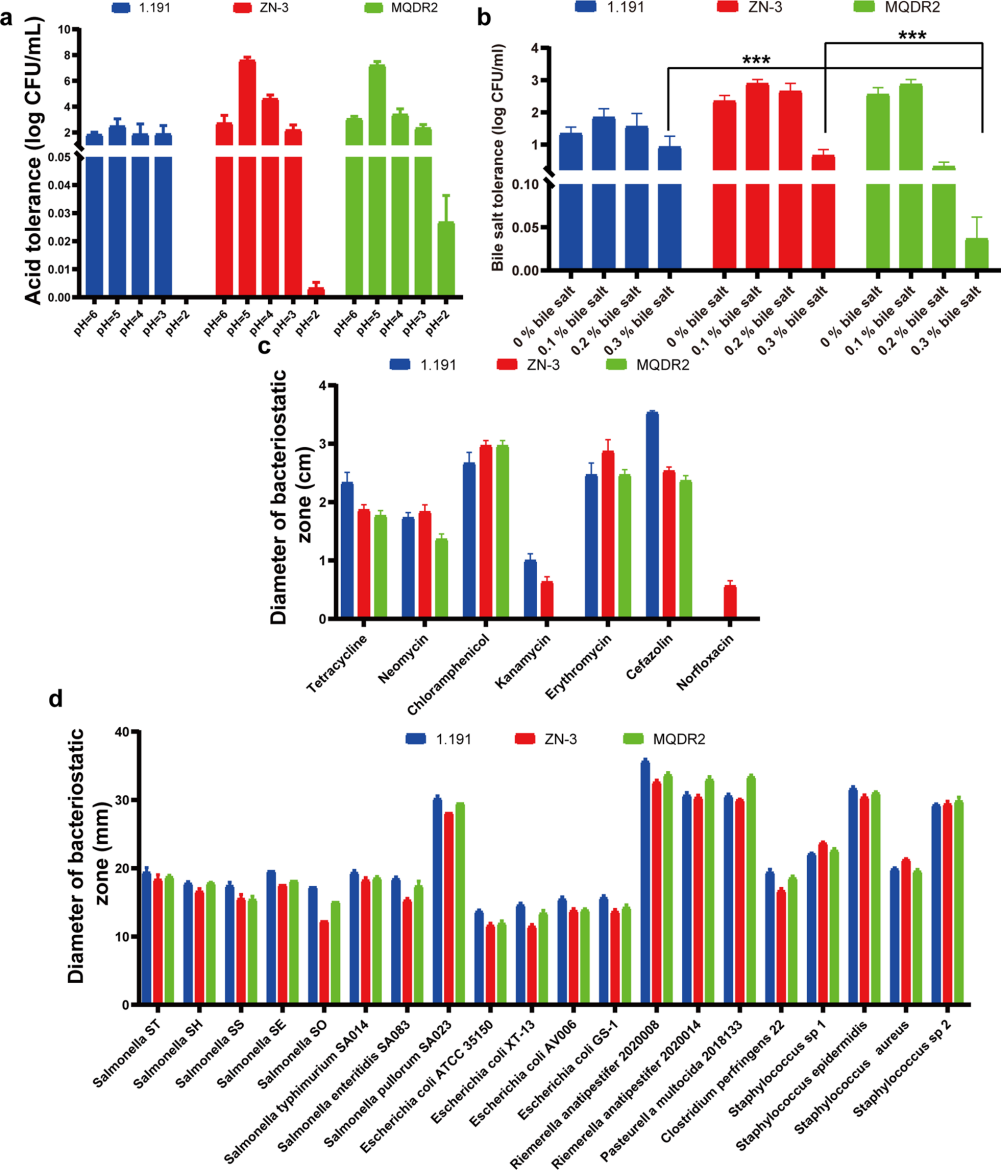
Probiotic properties of the three strains of *L. plantarum* isolated from the faecal samples of healthy pigs. **a** Effect of pH on the survival of the *L. plantarum* strains GG (log CFU/mL); **b** survival of the *L. plantarum* strains in the presence of different concentrations of oxgall (log CFU/mL) (*p < 0.05, **p < 0.01, ***p < 0.001); **c** minimum inhibitory concentration (μg/mL) of antibiotics for the *L. plantarum* strains; **d** the antimicrobial activity of the *L. plantarum* strains against potential pathogenic bacteria

The effects of simulated gastric juice on the survival rate of the isolated *L. plantarum* at an incubation time of 16 h are shown in Fig. 3a. In this study, the isolates were evaluated for tolerance to varying pH (2.0, 3.0, 4.0, 5.0, and 6.0). *L. plantarum* isolates showed negligible growth up to pH 4. However, at pH 5–6, an increase in growth rate was observed, above which the growth rate declined. Both the *L. plantarum* strains 1.191 and ZN-3 decreased in the culture medium of pH 2.0 but the viability of MQDR2 remained viable. Though the viability decreased slightly, but the *L. plantarum* strains ZN-3 survived in the culture medium of pH 2.0. Among all the isolates, MQDR2 showed the most significant results.

Bile salt tolerance is an essential factor determining the stability of probiotics in the intestine. Bile salts present in the intestinal tract disrupt the bacterial cell membrane and prevent them from entering the stomach. The maximum ZN-3 growth was observed in the presence of bile salts up to 0.3% (Fig. 3b). The isolates, however, were less tolerant to high concentrations, such as 0.6 and 1% (data not shown). Overall, in the presence of bile salts up to 0.3%, ZN-3 produced significantly higher biomass (p < 0.05) than that obtained with 1.191 or MQDR2.

### Antibiotic susceptibility

All isolates were analysed for their tolerance to antibiotics owing to safety considerations regarding the threat of antibiotic resistance in bacteria. The antibiotic susceptibility test was performed using the disc diffusion method by measuring the zone of inhibition for various antibiotics (Fig. 3c). The *L. plantarum* isolates were sensitive to cefazolin, tetracycline, neomycin, erythromycin, and chloramphenicol; among all the isolates, strain 1.191 showed the maximum diameter for the zone of inhibition against cefazolin (35.1 mm). It was concluded that isolates 1.191 and MQDR2 were resistant to norfloxacin. Antibiotic susceptibility tests showed that, compared to 1.191 and MQDR2, Z N-3 had better probiotic properties and was sensitive to all antibiotics used in this study.

### Antibacterial activities of *Lactobacillus s*trains against gram-positive and gram-negative pathogens

The isolated *Lactobacillus* strains showed notable antibacterial activity against all target bacteria commonly associated with gastrointestinal diseases. All three isolates successfully inhibited the growth of the clinical isolates *Salmonella* ST, *Salmonella* SH, *Salmonella* SS, *Salmonella* SE, *Salmonella* SO, *Salmonella typhimurium* SA014, *Salmonella enteritidis* SA083, *Salmonella pullorum* SA023, *Escherichia coli* (ATCC 35,150), *Escherichia coli* XT-13, *Escherichia coli* AV006, *Escherichia coli* GS-1, *Riemerella anatipestifer* 2,020,008, *Riemerella anatipestifer* 2,020,014, *Pasteurella multocida* 2,018,133, *Clostridium perfringens* 22, *Staphylococcus* sp 1, *Staphylococcus epidermidis*, *Staphylococcus aureus*, and *Staphylococcus* sp 2 (Fig. 3d). The diameters of the growth inhibition zones were similar among all the isolates.

### *L. plantarum* adheres to Caco-2 cells

**Fig. 4:**
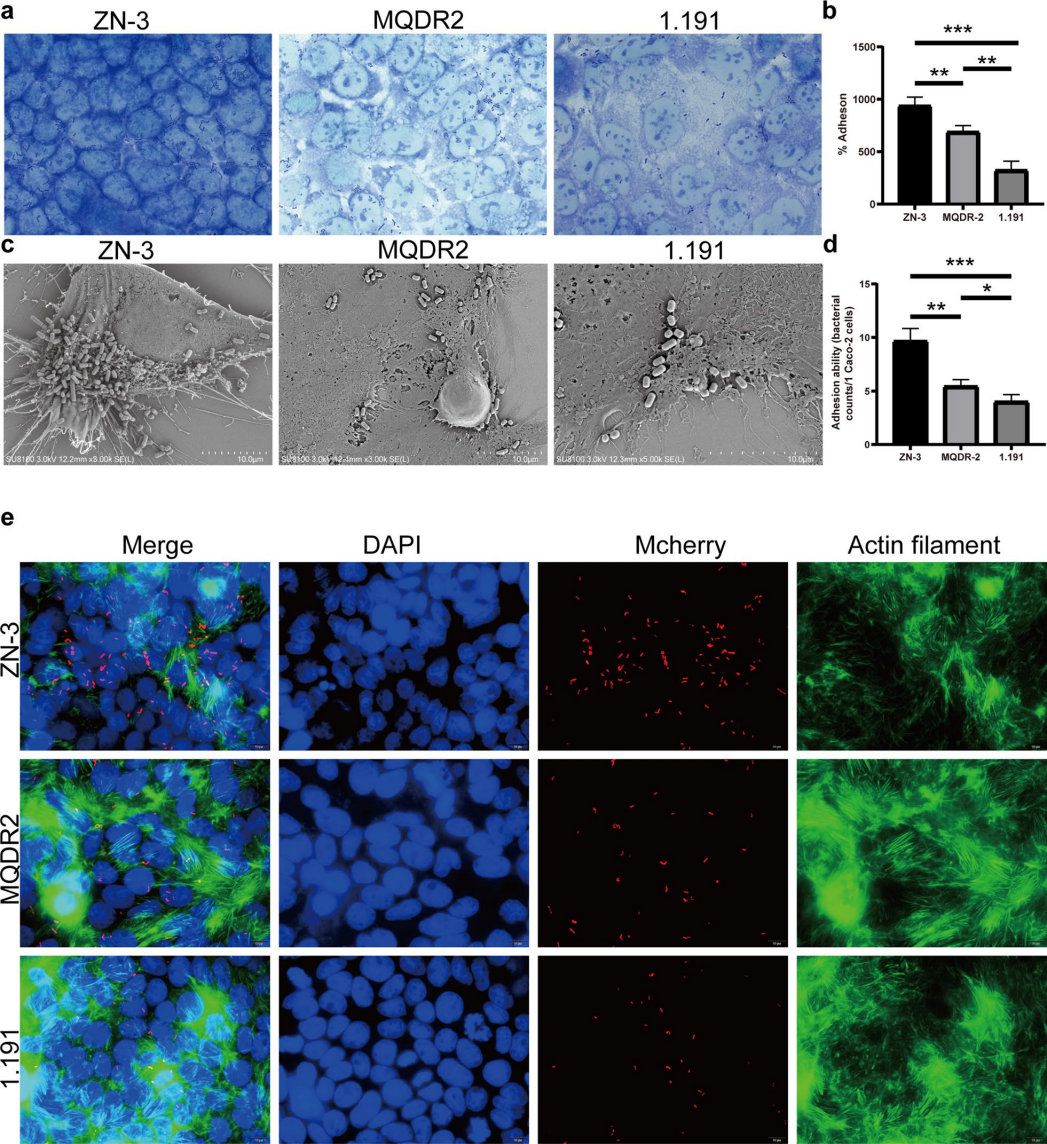
Adhesion efficiency of three *L. plantarum* strains to Caco-2 Cells. **a** Adhesion of representative *L. plantarum* strains on Caco-2 cell cultures observed under a microscope (magnification: 400×); **b** adhesion efficiency of *L. plantarum* strains in Caco-2 cells (*p < 0.05, **p < 0.01, ***p < 0.001). Values are expressed as mean ± standard error of mean; **c** examination of the adherence of *L. plantarum* strains to Caco-2 cells using scanning electron microscopy (SEM); **d** computational adhesion ability of the *L. plantarum* strains to Caco-2 cells using SEM (*p < 0.05, **p < 0.01, ***p < 0.001); **e** Epifluorescence microscopy (magnification: 400 ×) analysis of the adhesion efficiency of Mcherry-tagged *L. plantarum* strains to Caco-2 cells

The most important feature of any potential probiotic strain is its ability to adhere, which is referred to as the gold standard for identifying probiotic bacteria [42]. The adherence ability of the isolated ZN-3, 1.191, and MQDR2 to the Caco-2 cell line was determined by direct microscopic examination using Giemsa staining. All isolates were categorised as strongly adhesive (Fig. 4a). The adherent bacteria were further quantitated using a classical culture-dependent method. The adhesion efficiency was calculated as the percentage of adhesion values compared to the initial bacteria seeded in each well and assumed to be equal to 100%, confirming the high colonisation efficiency previously observed using microscopy. Both the *L. plantarum* strains ZN-3 and MQDR2 showed adhesion efficiency to Caco-2 cells, with an adhesion percentage ranging from 68–95% (Fig. 4b).

These isolates were further analysed using scanning electron microscopy (SEM), to understand the morphology of the isolates that adhered to Caco-2 cells. Both untreated and bacteria-treated Caco-2 cells were observed under an SEM (Fig. 4c). Figure 3c, at 2000× magnification, shows the presence of rod-shaped *Lactobacillus* adhering to the surface of Caco-2 cells. Among the three strains, ZN-3 exhibited significantly increased adherence to Caco-2 cells than those of the other strains (bacterial counts/cell, Fig. 4d). Immunofluorescence microscopy was used to visualise the adhesion ability of red fluorescent protein-expressing *L. plantarum* (ZN-3-mcherry, 1.191-mcherry, and MQDR2-mcherry). ZN-3-mcherry was the most adherent strain (Fig. 4e), and isolates 1.191 and MQDR2 exhibited moderate binding (Fig. 4e).

### Ex vivo epifluorescence time-course imaging of mouse intestine following the oral administration of IRFP713-expressing bacteria

**Fig. 5:**
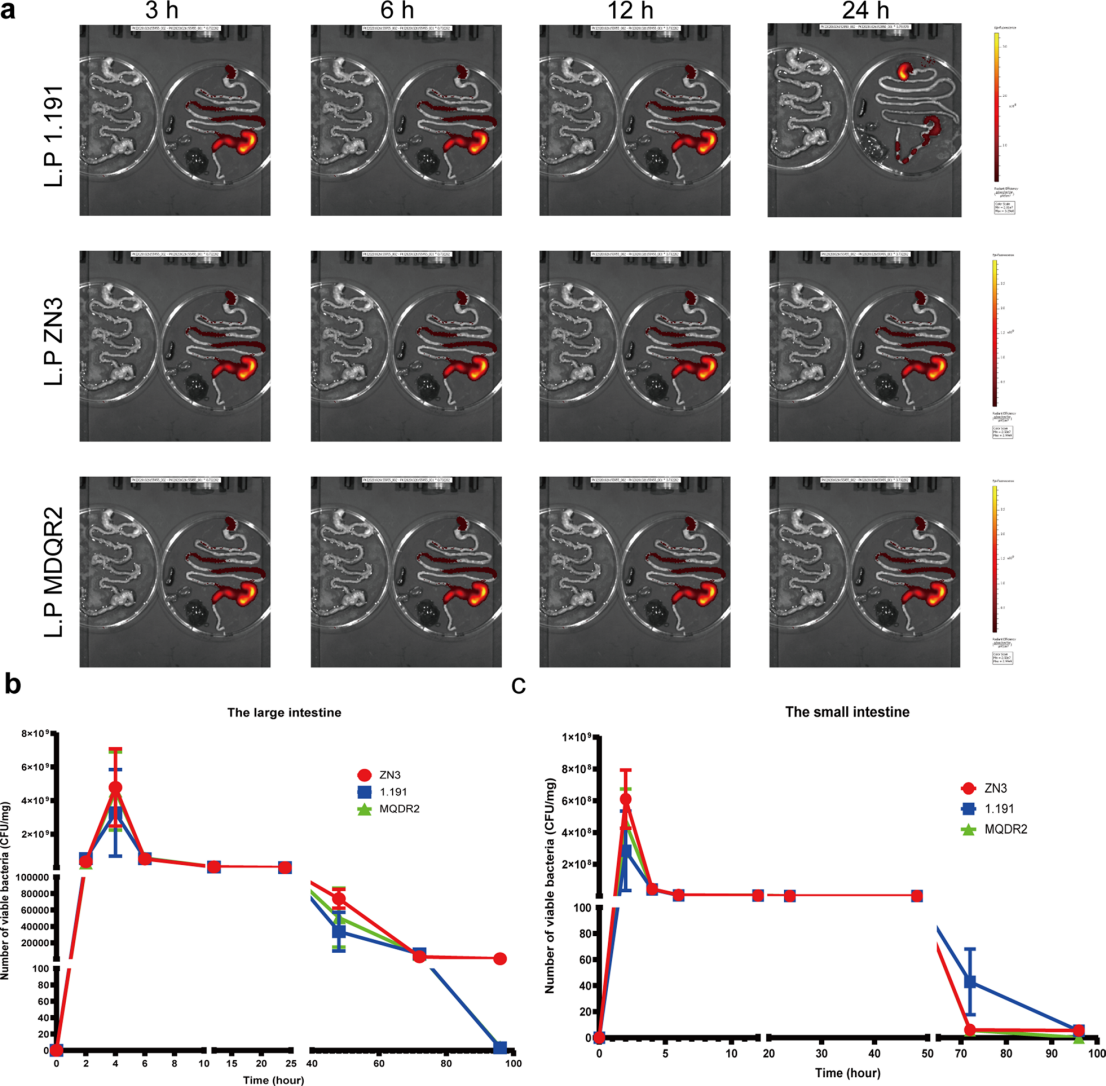
Transit and survival of IRFP713-expressing three *L. plantarum* strains in mice. Mice were administered 5.0 × 10^11^ cells. **a** The intestines were extracted at different time points and analysed using epifluorescence imaging; **b** and **c** The number of viable bacteria (CFU/mg; c) in different parts of the intestine were determined as a function of time

To determine the spatial and temporal transit of ZN-3-IRFP713, 1.191-IRFP713, and MQDR2-IRFP713 in the GIT of mice after a single oral administration, the intestines of three mice were removed at different time points and imaged ex vivo, respectively. The results showed that 180 min after the administration of the bacterial strains, ZN-3-IRFP713, 1.191-IRFP713, and MQDR2-IRFP713 survived passage through the stomach and IRFP713 signal cells were observed throughout the small intestine (Fig. 5a). After approximately 6 h, majority of the ZN-3-IRFP713, 1.191-IRFP713, and MQDR2-IRFP713 isolates had travelled through the small intestine and were located exclusively in the caecum and colon. Some of the viable cells of both strains remained in the small intestine after 6 h but emitted weak or no epifluorescence signals. They were retained in the caecum for several hours. Following this, they gradually cleared to the large intestine, from which more than 90% were secreted in 10 h, as observed by the decrease in both, CFU number and radiant efficiency. After 24 h, IRFP713 signal cells remained localised in the caecum and colon.

We monitored the number of ZN-3-IRFP713, 1.191-IRFP713, and MQDR2-IRFP713 bacteria, as well as their respective IRFP713 signals in the mice small and large intestines at different time points after the oral administration of bacteria (Fig. 5b and c). The number of viable bacteria increased with time in the small and large intestine, reaching its maximum level after 4 h with approximately 10^9^ CFU/100 mg intestinal content for each strain (Fig. 5b and c). The abundance of ZN-3-IRFP713, 1.191-IRFP713, and MQDR2-IRFP713 in mice small and large intestine plateaued for approximately 4 h and then declined. After 24 h, this number reached approximately 10^5^ CFU/100 mg intestinal content. After 96 h, no *L. plantarum*-IRFP713 was found in the intestinal contents, whereas the number of *L. plantarum*-IRFP713 organisms was still approximately 10^2^ CFU/100 mg intestinal contents. In summary, these results indicate that the three *Lactobacillus* strains are capable of colonising the intestines of mice, and the ZN-3 strain had the strongest ability to colonise and persist in the intestines of mice.

From these studies, we have a better understanding of probiotic candidates with a positive effect Lactobacillus plantarum strains and provide guidance for using genome engineering to consider as vaccine delivery vehicles. We finally selected ZN-3 as the research object. Please see Additional file 4: Table S1 for scoring criteria.

### *L. plantarum* displayed H1N1 HA1 protein on the cell surface

**Fig. 6:**
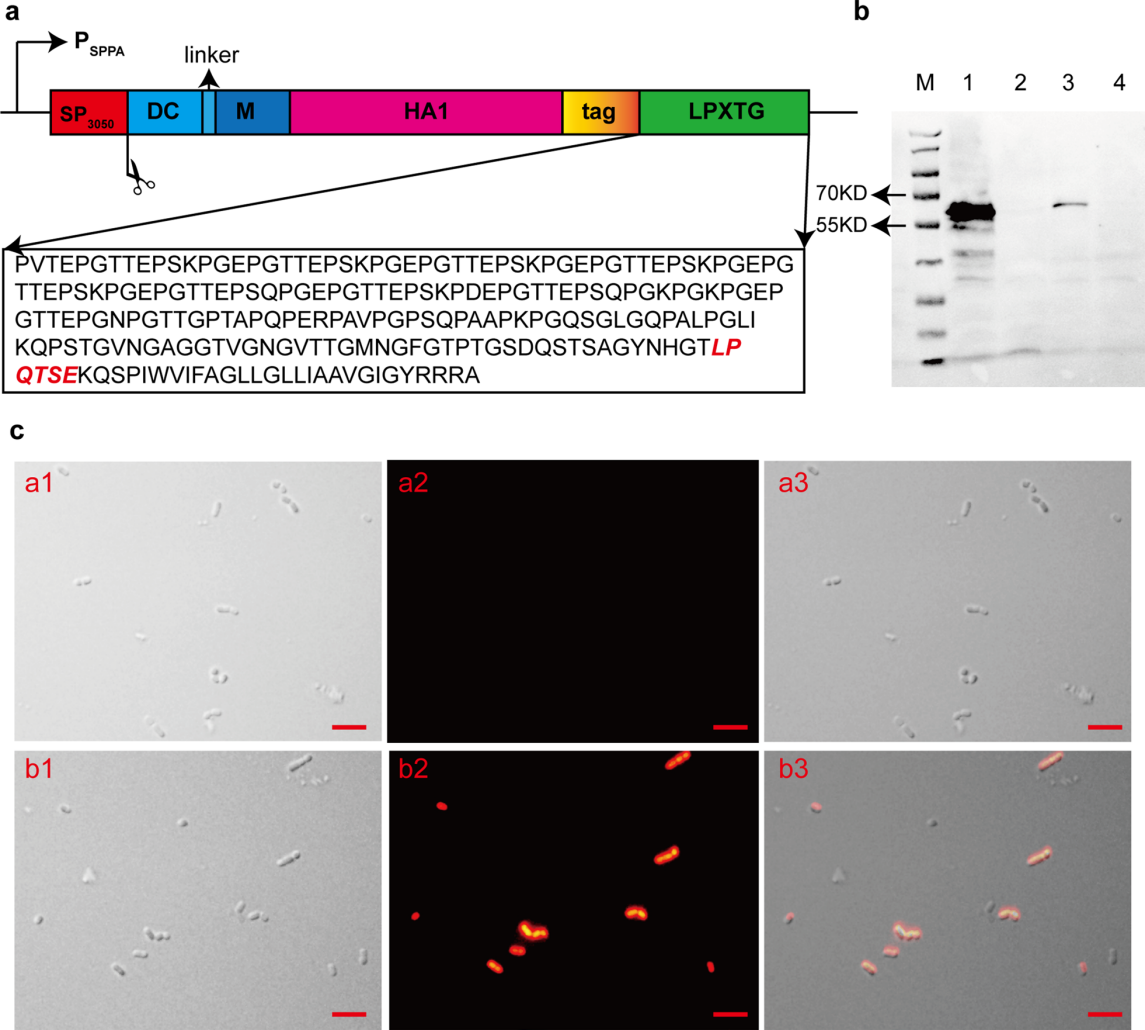
Expression of HA1 presented on the *L. plantarum* ZN-3 surface. **a** Schematic of pSIP401-SP1216-HA1-2578. A GS linker was inserted between DCpep and M cell-targeting peptide; **b** the protein of interest was identified on western blot. A relevant immunoreactive band was produced by the cell precipitation of strain pSIP401-HA1-ZN-3 (lane 1) and total cell supernatant of strain pSIP401-HA1-ZN-3(lane 3), but not strain pSIP401-ZN-3 (lanes 2 and 4). M: protein molecular weight marker; **c** Immunofluorescence microscopy pSIP401-ZN-3 (a1, a2, and a3) and pSIP401-HA1-ZN-3 (b1, b2, and b3) (magnification: 1000 ×)

To improve vaccination efficiency, an oral DC-targeted and M-targeted mucosal vaccine using *L. plantarum* as a vector delivering the HA1 protein fused to DCpep and the M cell-targeting peptide was constructed. The gene sequences encoding recombinant HA1 proteins were successfully cloned into the pSIP401 expression vector (Fig. 6a). Sequencing of the plasmids confirmed the presence of the corresponding gene sequences in the frame with the SP1216 and 2578 domains. Subsequently, *L. plantarum* strain ZN-3 cells were transformed with recombinant plasmids (pSIP401-SP1216-HA1-2578) to engineer *L. plantarum* strains (pSIP401-HA1-ZN-3) expressing recombinant HA1 proteins. Western blot analysis of cell fractions showed the presence of specific immunoblots in the cell wall fraction at positions corresponding to the sizes of the recombinant fusion proteins (Fig. 6b). Immunostaining with FITC-labelled secondary antibody resulted in strong immunofluorescence of the engineered *L. plantarum* cells induced with the peptide pheromone IP-673, indicating the specific binding ability of the antibody to the recombinant HA1 protein surface displayed by the engineered *L. plantarum* cells only. No detectable signal was observed in uninduced *L. plantarum* cells (Fig. 6c). These results indicated the precise expression and location of the antigen proteins.

### HA-specific IgG and sIgA responses after oral administration and intranasal immunization

**Fig. 7:**
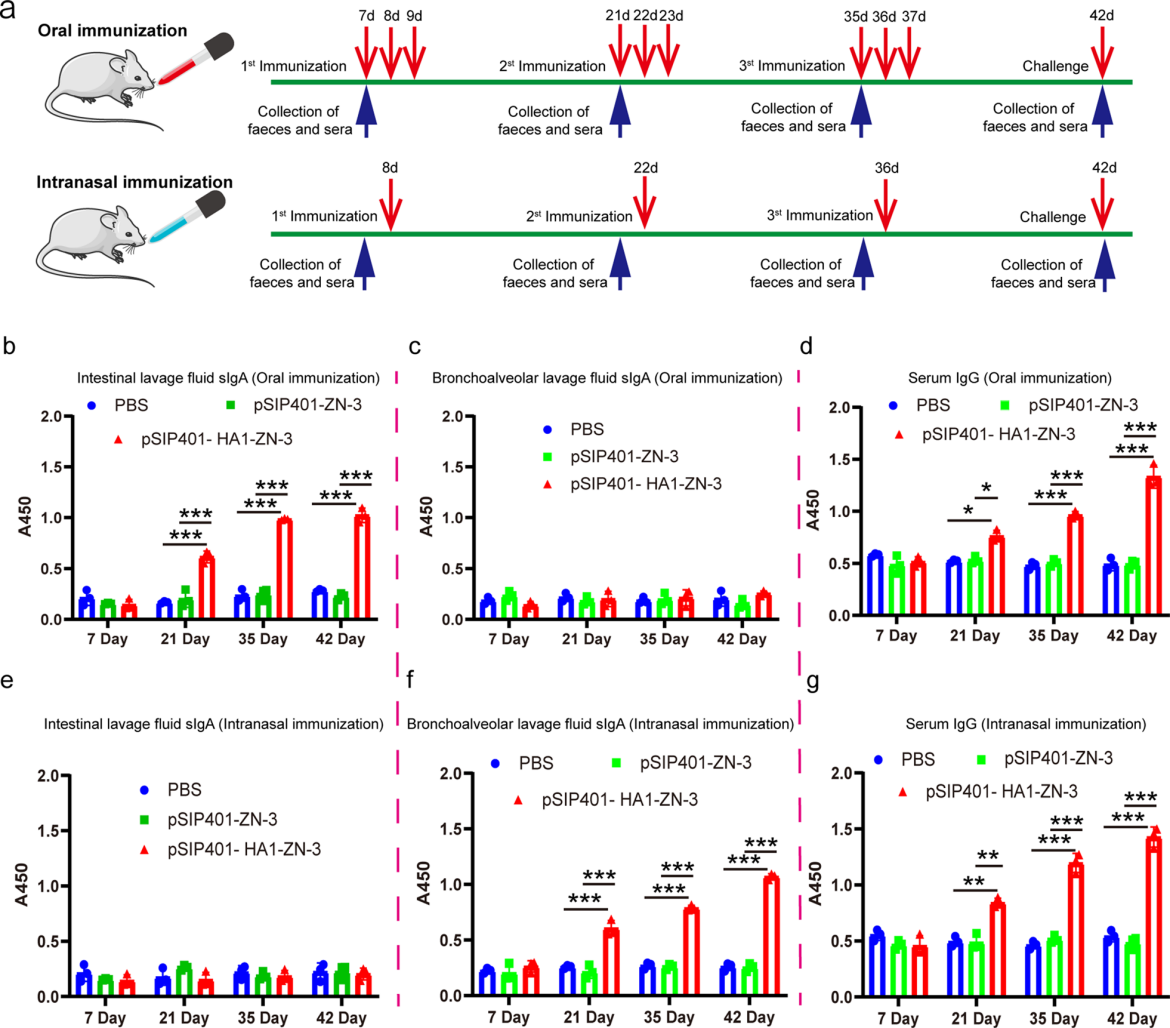
Specific anti-HA1 antibody levels in mice orally and intranasally immunized with the engineered *L. plantarum* strains. **a** Experimental protocol for oral and intranasal immunisation of mice with pSIP401-ZN-3 and pSIP401-HA1-ZN-3; **b, e** Specific secretory immunoglobulin A (sIgA) levels in intestinal lavage fluid from mice in each group after immunisation with recombinant *L. plantarum* strains; **c**, **f** Specific sIgA levels in bronchoalveolar lavage fluid from mice in each group after immunisation with recombinant *L. plantarum* strains; **d**, **g** Specific serum immunoglobulin G (IgG) levels in mice from each group after immunisation with recombinant *L. plantarum* strains. *P < 0.05, **P < 0.01, **P < 0.001 were defined as statistically significant values

We tested whether the recombinant HA1 proteins could increase the Ag-specific antibody responses induced by oral administration and intranasal immunisation of engineered *L. plantarum* strains pSIP401-HA1-ZN-3. BALB/c mice were orally and intranasally immunised with pSIP401-HA1-ZN-3 or pSIP401-ZN-3 using three bi-weekly inoculations, each consisting of three daily doses (Fig. 7a). The development of anti-HA1 antibody responses in the gut and circulation was monitored over time as HA1-specific ILF IgA and serum IgG, respectively. The ILF HA1-specific IgA was significantly induced by day 21 in mice administered pSIP401-HA1-ZN-3 (Fig. 7b). The ELISA results showed that oral immunisation with pSIP401-HA1-ZN-3 did not produce a significantly higher sIgA level in BALF than that in the pSIP401-ZN-3 and PBS groups throughout the experiment (Fig. 7c). Serum nuclease-specific IgG levels increased by day 22 in mice administered pSIP401-HA1-ZN-3, and these levels were further elevated by day 42 (Fig. 7d).

Although intranasal immunisation with pSIP401-HA1-ZN-3 increased the sIgA level in ILF compared to that in the control mice, no significant difference was observed in the groups administered only pSIP401-ZN-3 or PBS (Fig. 7e). The mean ELISA level of sIgA in the BALF collected from mice belonging to the intranasal immunisation groups suggests a significant increase in sIgA levels in the mice administered induced engineered *L. plantarum* cells expressing HA1 than those in the control mice (P < 0.0001) (Fig. 7f). In the case of serum IgG response, antibody levels were found only at the basal level. Consistent with BALF, the mean serum IgG level was significantly higher in the mice intranasally administered with pSIP401-HA1-ZN-3 cells than that in the control mice (P < 0.0001) (Fig. 7g). These results indicated that, pSIP401-HA1-ZN-3 is effective in activating local mucosal immune response.

**Fig. 8:**
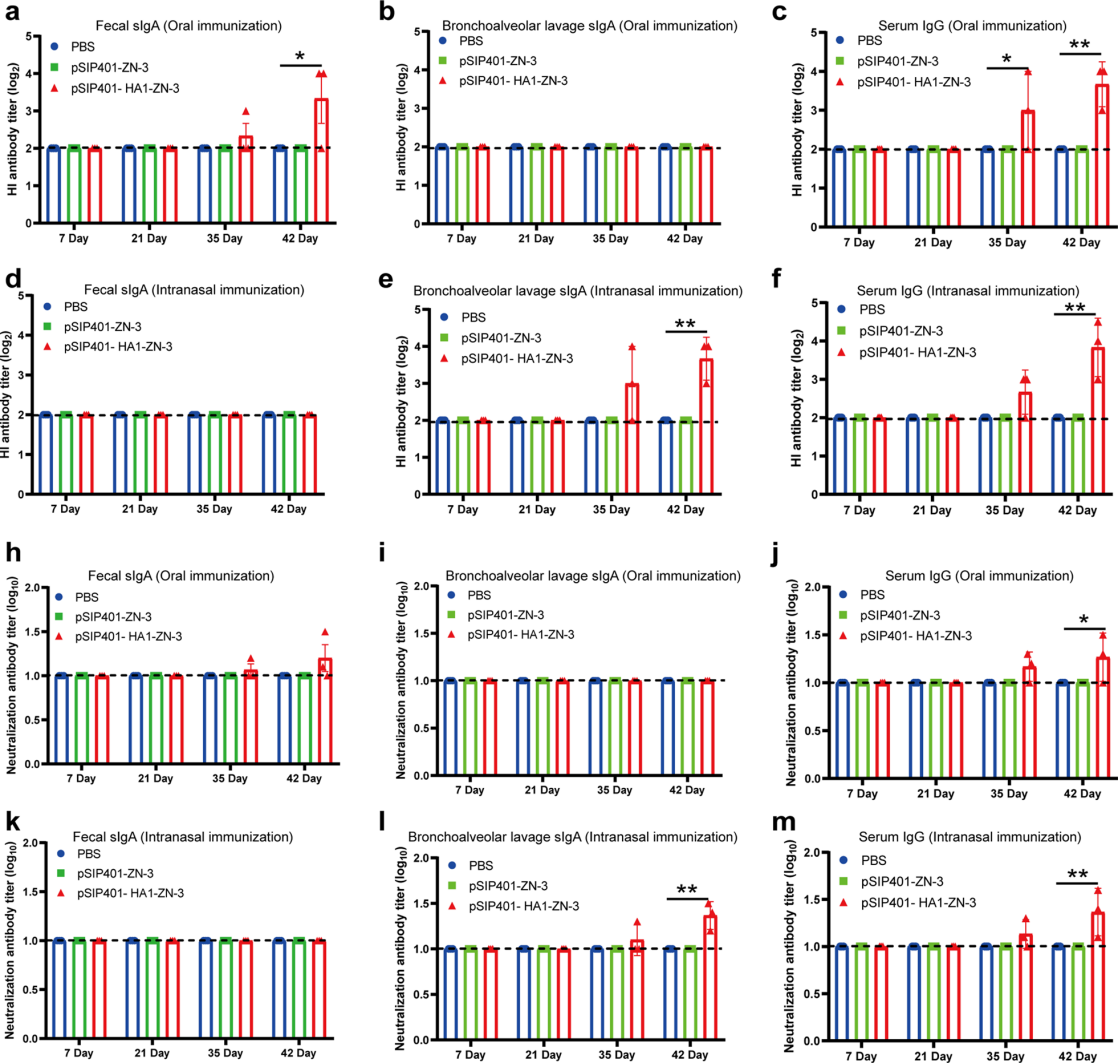
HI and VN antibody titers in mice after immunization with the engineered *L. plantarum* strains. Bronchoalveolar lavage fluid (BALF), intestinal lavage fluid (ILF) and sera were collected for 1 week after the third immunization for HI, VN antibody titers detection. **a**–**f** HI antibody titers was determined with swIAV H1N1 virus, the results were calculate by value of log_2_. **h**–**m** The VN antibody titers were determined against 100 TCID_50_ swIAV H1N1 virus, the results were calculated by value of log_10_ finally. Significant differences of vaccination with univalent inactivated vaccine group were recorded (*p < 0.05, **P < 0.01, ***P < 0.001)

To determine whether the antibody had the protective effect against swIAV H1N1 virus, we detected the levels of antibodies against swIAV H1N1 virus in sera of mice by Hemagglutination inhibition (HI) assay and the virus neutralization (VN) assay (Fig. 8). HI antibody titers of 3.3 log_2_ were detected from the ILF of oral immunisation with pSIP401-HA1-ZN-3 group against H1N1 virus at 1 week after the third immunization (Fig. 8a). In addition, HI antibody titers of 3.6 log_2_ were detected from the BALF of intranasal immunisation with pSIP401-HA1-ZN-3 group against H1N1 virus at 1 week after the third immunization (Fig. 8d). All PBS-immunized and pSIP401-ZN-3 group mice were seronegative, whereas the HI titers in oral immunisation and intranasal immunisation with pSIP401-HA1-ZN-3 groups increase over time, with reaching highest level at 3.6 log_2_ and 3.8 log_2_ respectively at 1 week after the third immunization (Fig. 8d and f). The VN antibody titers against H1N1 viruses were also determined respectively (Fig. 8h - m). Overall, the standard measures of influenza vaccine VN antibody titers were weak regardless of the recombinant Lactobacillus plantarum vaccine used in mice. In addition to the above, we also detected the levels of antibodies against swIAV H3N2 and AIV H5N6 virus in sera of mice by Hemagglutination inhibition (HI) assay and the virus neutralization (VN) assay (Additional file 2: Fig. S2 and Additional file 3: Fig. S3).

### Systemic cell-mediated immune responses to recombinant *L. plantarum* immunization

**Fig. 9:**
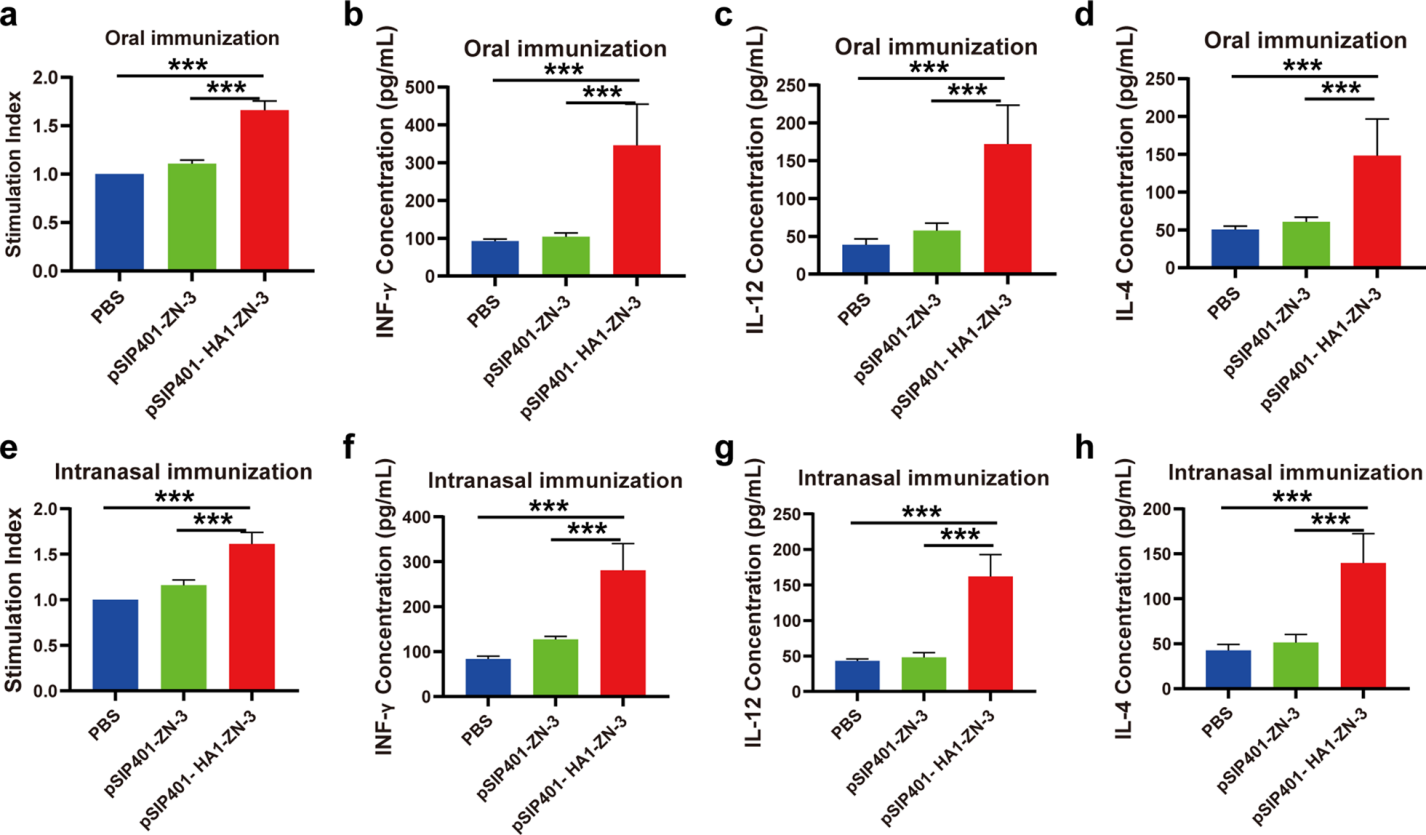
Assessment of lymphoproliferative responses and comparison of cytokine profiles in splenocyte culture supernatants restimulated with recombinant H1N1 HA1 antigen. **a**, **e** Three mice in each group were sacrificed 7 days after the third immunization, and splenocytes proliferation in response to antigen stimulation was estimated using an MTT assay. Values are presented as the mean ± SD for three samples. Differences between all immunized and control groups were statistically highly significant (p < 0.001). **b**–**d** and **f**–**h** The ELISA results are representative of three independent experiments and are expressed as the mean ± SD. One-way ANOVA was used to analyze differences among groups. ***, P < 0.001; **, p < 0.01. The graphs also show a statistically significant difference between all vaccinated and control groups (P < 0.001)

We evaluated the proliferative response and cytokine secretion of splenic lymphocytes primed with the HA1 antigen in vitro to determine the systemic cell-mediated immune responses to recombinant *L. plantarum*. The splenocytes obtained from the mice in different groups on the 42d post vaccination were stimulated using the HA1 protein, followed by the determination of lymphocyte proliferation by MTT assay. As shown in Fig. 9a and b, the proliferation of spleen lymphocytes in mice orally immunized or intranasally immunized with pSIP401-HA1-ZN-3 increased significantly. As expected, mice in the groups treated with pSIP401-ZN-3 or PBS showed very little lymphoproliferative response. These results suggested that immunization with pSIP401-HA1-ZN-3 elicited a more robust splenocytic proliferative response against H1N1 HA1.

To further investigate the effect of pSIP401-HA1-ZN-3 on the polarization of the immune response, cytokine secretion profiles in splenocyte culture supernatants of immunized mice were analyzed by ELISA 1 week after the final immunization. Notably, Th1 cell differentiation is mainly induced by IFN-γ and IL-12, while Th2 bias is associated with cytokines such as IL-4. Cytokine ELISA with culture supernatants harvested at 72 h showed that in response to HA1, splenocytes from mice orally administered with the pSIP401-HA1-ZN-3 produced higher levels of the Th1-associated cytokine IFN-γ, IL-12 (Fig. 9c and e) and the Th2-associated cytokine IL-4 (Fig. 9g) compared to those in mice administered with pSIP401-ZN-3 or PBS. The same result was observed by intranasally administered with the pSIP401-HA1-ZN-3. As shown in Fig. 9d, f and h, splenocytes of the vaccine groups (pSIP401-HA1-ZN-3) in a response to antigenic stimulus expressed a notably higher level of IFN-γ, IL-12 and IL-4 as compared to control groups (pSIP401-ZN-3 and PBS; P < 0.001).

### Protection against lethal H1N1 virus challenge

**Fig. 10:**
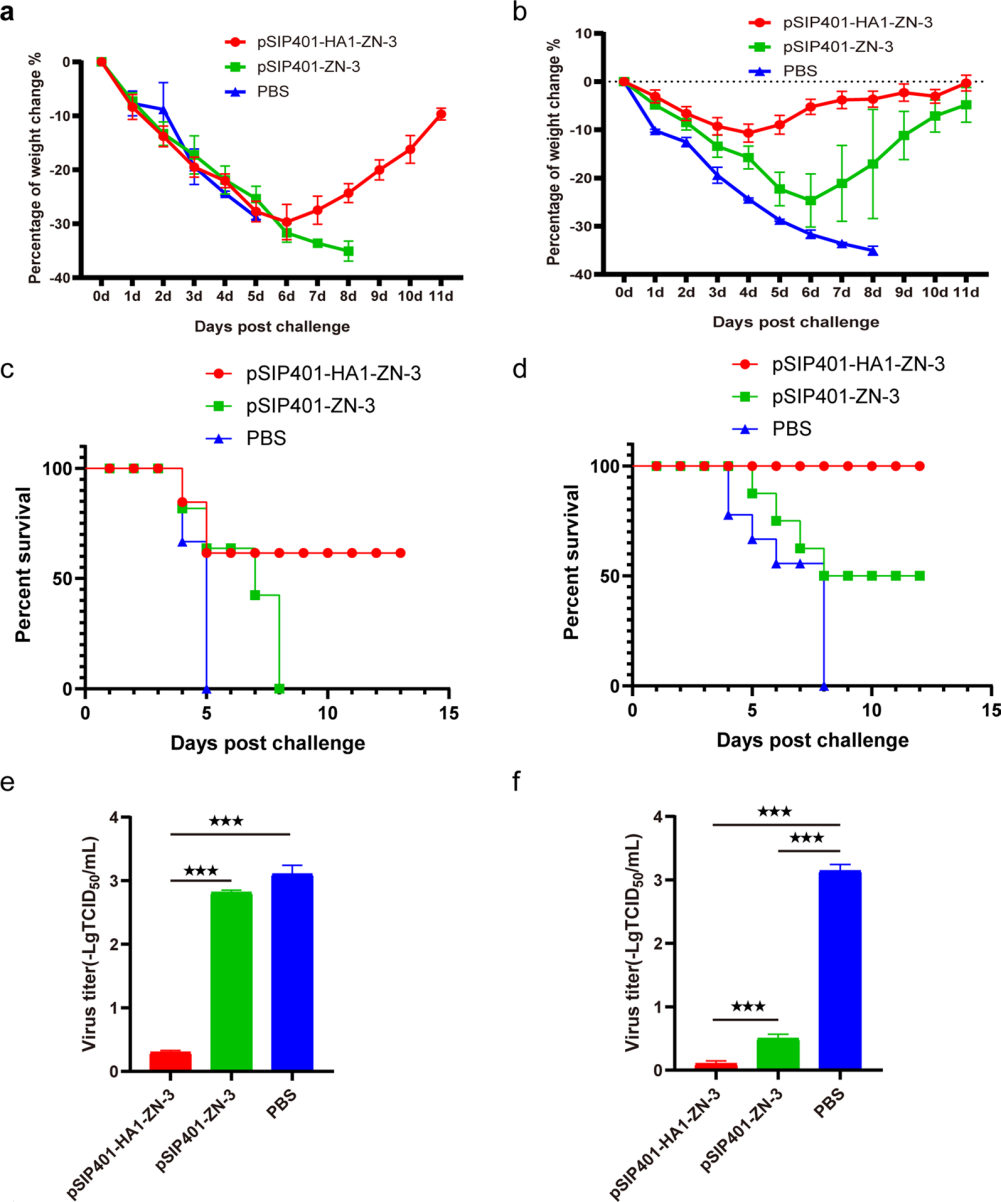
Changes in body weight and survival rate of mice following a lethal challenge with influenza virus. Seven days after the last immunisation, all mice in the study group were intranasally challenged with H1N1 virus. **a** Body weight and **c** survival of mice orally immunised with PBS, pSIP401-ZN-3, or pSIP401-HA1-ZN-3 were monitored daily for 15 days following virus challenge; **b** Body weight and **d** survival of mice intranasally immunised with PBS, pSIP401-ZN-3, or pSIP401-HA1-ZN-3 were monitored daily for 14 days following virus challenge. Virus titres in (**e**) the lungs of mice orally immunised with PBS, pSIP401-ZN-3, and pSIP401-HA1-ZN-3 at DPC 6, and in (**f**) the lungs of mice orally immunised with PBS, pSIP401-ZN-3, and pSIP401-HA1-ZN-3 at DPC 6 are shown. Asterisks indicate statistically significant differences compared with the PBS-treated group (*p < 0.05, **p < 0.01, ***p < 0.001). PBS: phosphate-buffered saline

A week after the final immunisation, the mice were intranasally challenged with lethal doses of highly pathogenic H1N1 virus and closely monitored for 14 days for weight loss and mortality. After viral challenge, all mice experienced certain levels of body weight loss (Fig. 10a and b); however, mice orally immunised with pSIP401-HA1-ZN-3 gradually recovered after 6 days with 60% survival (Fig. 10c). In addition, mice intranasal immunised with pSIP401-HA1-ZN-3 gradually recovered after 4 days with 100% survival (Fig. 10d). In contrast, control mice (PBS-treated) and mice orally immunised with the empty plasmid vector (pSIP401-ZN-3) died within 10 days of challenge. Interestingly, the protective efficacy was 50% for the mice intranasally immunized with pSIP401-ZN-3. The mice were challenged with H1N1 and the viral titres in the lungs of infected mice (n = 3/group) were determined 4 days post-challenge (DPC) (Fig. 10e). Mock-vaccinated (PBS) mice orally immunised with pSIP401-ZN-3 exhibited lung viral titres of approximately 5 × 10^3^ TCID_50_/mL on day 4 post-challenge, whereas mice intranasally immunised with pSIP401-HA1-ZN-3 showed no detectable virus in the lungs. Furthermore, at DPC 6, the challenge virus titre in the lungs was reduced in both pSIP401-ZN-3 and pSIP401-HA1-ZN-3 intranasally immunised mice groups compared to the mock-vaccinated mice, suggesting less replication/shedding in the lungs of vaccinated mice (Fig. 10f).

**Fig. 11:**
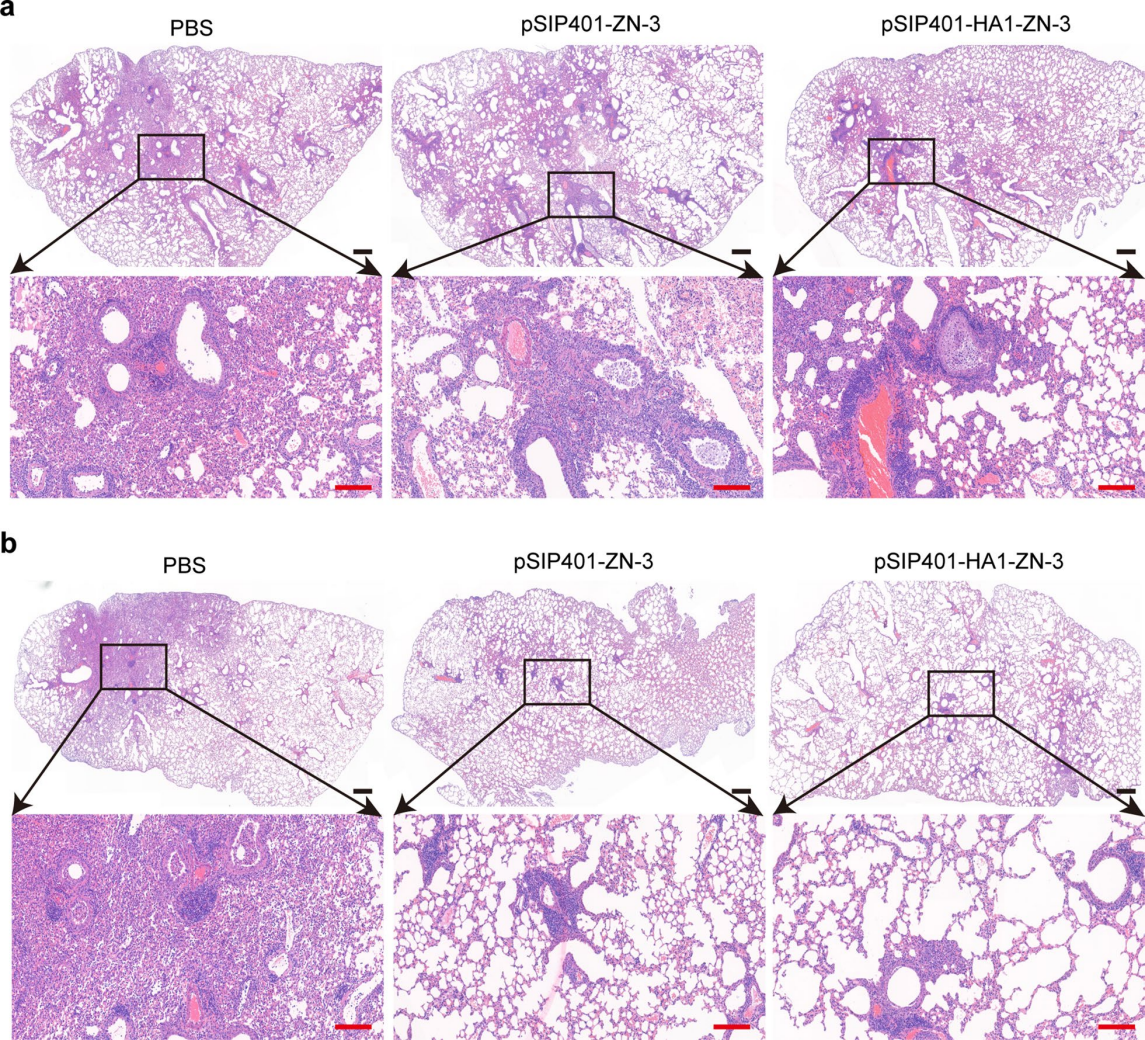
Histopathological lesions in the lungs of H1N1-challenged mice stained with haematoxylin and eosin (H&E). **a** H&E for oral administration group; **b** H&E for intranasal immunization group; Scale bar (black) 200 μm; (red) 50 μm. PBS: phosphate buffered saline

To determine whether the antigen-specific mucosal immunity in mice induced by pSIP401-HA1-ZN-3 resulted in pulmonary tissue damage upon H1N1 infection, we challenged vaccinated mice with H1N1 and performed histopathological analysis 7 DPC (Fig. 11). The H&E-stained lungs (Fig. 11a) showed significantly greater inflammation at 7 DPC in the PBS-inoculated group or oral immunisation with pSIP401-ZN-3 group than that with oral immunisation with pSIP401-HA1-ZN-3. In addition, at 7 DPC, inflammation was highest in PBS-treated mice, followed by intranasal immunisation with pSIP401-ZN-3, and lowest in intranasal immunisation with pSIP401-HA1-ZN-3 (Fig. 11b). These observations indicate that oral immunisation of mice with pSIP401-HA1-ZN-3 or intranasal immunisation with pSIP401-HA1-ZN-3 or pSIP401-ZN-3 provided protection against a lethal dose of the influenza virus.

**Fig. 12:**
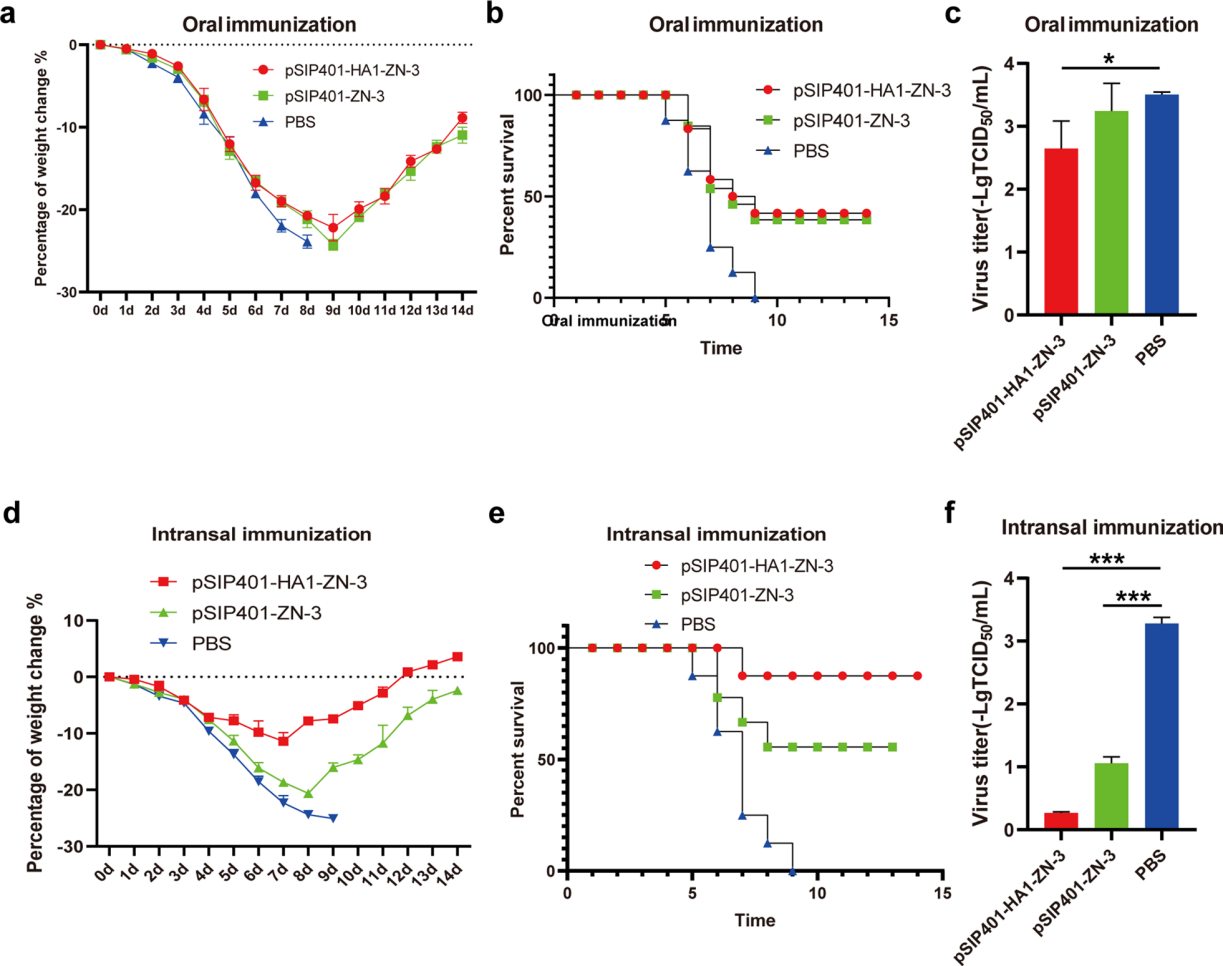
Weight changes and percent survival in mice after challenging with swIAV H3N2 virus. Groups of immunized and unvaccinated mice intranasally challenged with swIAV H3N2 virus at 1 week after the third immunization. Average body weight changes (**a**, **d**) and survival rates (**b**, **e**) were monitored for 14 days. Values indicate the mean weight changes of all of the mice in each group after virus challenge. The lungs of each mouse group were collected for viral titers (**c**, **f**) detection at 5 day post-challenge in MDCK cells and the results were calculated by value of log_10_. Significant difference of vaccination with DMEM non-inactivated vaccine group was recorded (*p < 0.05, **P < 0.01, ***P < 0.001)

Next, we followed up mice in the orally immunized or intranasally immunized with recombinant Lactobacillus plantarum vaccine vaccinated groups with a challenge study using heterologous live SIV H3N2 to determine if mice immunized against pSIP401-HA1-ZN-3 (H1N1) were protected against virulent H3N2. The mice orally immunised with pSIP401-HA1-ZN-3 and pSIP401-ZN-3 resulted in weight loss up to 23–25% and survival rates 41–46% after challenge with H3N2 virus whereas unvaccinated mice did not survive H3N2 virus infection (Fig. 12a and b). Nonetheless, we observed in the orally immunised with pSIP401-HA1-ZN-3 animals a reduced amount of virus in lung samples (Fig. 12c), compared to the mock-immunized mice. Taken together, we observed a reduced viral load of vaccinated animals after the different challenge infections. The intranasally immunised with pSIP401-HA1-ZN-3 group exhibited significant higher efficacy of protection against lethal H3N2 virus infection, as evidenced by less weight loss (~ 11.3%) and ~ 87.5% survival rates (Fig. 12d and e). Furthermore, the intranasally immunised with pSIP401-HA1-ZN-3 mice had significantly lower mean viral lung titers than the mock-vaccinated mice (Fig. 12f). These results suggest that the intranasally immunised with pSIP401-HA1-ZN-3 group conferred cross-protection against antigenically H3N2 influenza A viruses.

## Discussion

Because of their susceptibility to avian, swine, and human IAVs, pigs are regarded as a mixing vessel for generating novel reassortant influenza viruses capable of replicating and spreading among humans [43–45]. Swine influenza viruses, as a zoonotic disease, has an important impact on public health [46]. G4 genotype reassortant Eurasian avian-like (EA) H1N1 virus possessing possessing 2009 pandemic (pdm/09) and triple-reassortant (TR)-derived internal genes has replaced the G1 genotype EA H1N1 virus and has become predominant in swine populations in China [6, 45, 47]. To protect the human population and animals from precarious yearly epidemics and from the potential threat of new pandemics, only a broad-spectrum, efficient and non-adverse-effect vaccine should be qualified [48]. A recombinant mucosal vaccine may be the best choice to fulfill all of these criteria. Accordingly, we constructed a recombinant lactobacillus strains pSIP401-HA1-ZN-3, that produce the H1N1 virus protective antigen HA1. Oral immunization and intranasal immunization with the recombinant lactobacilli elicited HA1-specific antibodies and Th1-type cellular immune responses.

In this study, six L. plantarum strains isolated from faecal samples of healthy pigs and strains ZN-3, 1.191, and MQDR2 were confirmed to achieve high expression levels of target genes and proteins. Thus, ZN-3, 1.191, and MQDR2 are promising carriers for gene transfer and vaccine delivery. One of the most important criteria for identifying a potential probiotic is its ability to cope with acidic conditions and bile salts, which dictates its survival in the extreme environment of the GIT [49]. The acid and bile tolerance results indicate that the three L. plantarum strains of ZN-3, 1.191, and MQDR2 could survive the study conditions, suggesting that the three strains could pass through the stomach and function effectively.

Inhibiting the growth of harmful bacteria is a major property of probiotics. In this study, we found that strains ZN-3, 1.191, and MQDR2 inhibited the growth of a common intestinal pathogen in a commercial pig farm based on inhibition zone assays. This antagonistic activity has mainly been ascribed to the production of antimicrobial substances or to the different metabolites produced by probiotic strains [49]. According to the defined priority scale for probiotic selection [50], a key evaluation criterion is the adhesion efficiency to the intestinal epithelium aimed at ensuring persistence in the gut environment and thus conferring beneficial effects to human and animal health [51]. In this study, Caco-2 cells, which have been used as an in vitro model for the intestinal epithelium, were used to assess the adhesion ability of the isolated strains. The adherence of ZN-3 was almost twice that of 1.191 and MQDR2. Similar results were obtained using SEM analysis, to determine the in vitro epithelial cell adherence effect. Different imaging modalities have been employed to monitor the fate of bacteria after administration in mice [33]. The current study was designed to investigate the colonisation (spatial and temporal) dynamics of L. plantarum ZN-3-IRFP713, 1.191-IRFP713, and MQDR2-IRFP713 orally inoculated once over a period of 24 h. In conclusion, the caecum and colon were the predominant sites for persistent L. plantarum infection in mice.

Since influenza viruses naturally infect the mucosa, it is highly desirable to develop vaccines that can induce mucosal as well as systemic immune responses [52]. The mucosa is the first physical barrier restricting the entry of pathogenic microorganisms into animals, and the mucosal immune system provides primary protection against microbial infection. SIgA is an important component of mucosal immunity. It is the most abundant immunoglobulin isotype in human secretions [53] and effectively protects against influenza virus infections [54]. It has been reported to be more important than IgG in protecting the upper respiratory tract, specifically the nose and trachea [54, 55], primarily by reducing viral attachment and preventing virus internalisation at the mucosal surfaces, thereby preventing infection [54, 56–58]. In this study, L. plantarum ZN-3 was used as a vehicle for the delivery of truncated H1N1 HA1 antigen.

It is generally assumed that IgA is the main antibody isotype and effector in the host defense at mucosal surfaces [59]. Our results indicated that pSIP401-HA1-ZN-3, expressing HA1, triggered a mucosal immune response in both the respiratory and digestive tracts. The recombinant pSIP401-HA1-ZN-3 bacteria also effectively triggered systemic immune responses against HA1, although the HA1-specific IgG and HI antibody titers were low. Our studies demonstrate that mucosal immunization with recombinant lactobacilli can elicit both mucosal IgA and circulating IgG and support the theory that mucosal immunization can provoke both mucosal and circulating antibody responses. These results are consistent with other observations [59, 60]. Although oral immunization and intranasal immunization with recombinant pSIP401-HA1-ZN-3 induced HA1-specific serum IgG and mucosal IgA production, cell-mediated immune responses also played a crucial role in protecting the host from invading pathogens. Our results demonstrated that the purified HA1 protein obviously promoted the splenic lymphocyte proliferative reaction and cytokine secretion compared with those of controls (P < 0.001). Our study showed that Th1-type (IFN-γ) and Th2-type (IL-4) cytokine responses specific to HA1 were significantly induced, which may have contributed to broadening the protection efficiency of our vaccine candidate pSIP401-HA1-ZN-3 against divergent influenza subtypes.

In the present study, immunization with pSIP401-HA1-ZN-3 significantly increased the specific sIgA response than that in the non-immunised group. Furthermore, HA1-specific sIgA in faecal extracts demonstrated that oral immunisation with pSIP401-HA1-ZN-3 in mice elicited significant mucosal immunity in the GIT. Therefore, in this study, sIgA was not detected in tracheal mucosal; however, 60% mice were protected against viral challenge. One advantage of intranasal immunisation is its potential to induce a mucosal immune response. In the present study, mice vaccinated intranasally with pSIP401-HA1-ZN-3 elicited strong mucosal immune responses, such as the secretion of IgA in the respiratory mucosa. Final immune protection was assessed using the homologous H1N1 virus challenge monitoring after post-challenge indicated no significant decrease in the body weight of mice vaccinated with pSIP401-HA1-ZN-3, and the mortality rate results revealed that pSIP401-HA1-ZN-3 could provide 100% protection efficacy against homologous H1N1 virus. Pathological analysis revealed that the L. plantarum ZN-3 expressing H1N1 HA1 protein provided protection against pathological injury of influenza virus target organs. After heterosubtypic challenge with H3N2, we detected a considerably decreased viral load in the lungs of intranasally immunized animals along with an less weight loss (~ 11.3%) and ~ 87.5% survival rates. We speculate that these findings indicate systemic and local cellular immune responses triggered by vaccination. However, as weight change and viral load did not differ between orally immunized and control mice, we must conclude that this orally immunized vaccine formulation did not cross protect against experimental heterologous H3N2 challenge.

## Conclusions

This study shows the feasibility of inducing protective humoral and mucosal immunity after oral and intranasal administration of pSIP401-HA1-ZN-3 without the use of an adjuvant. In summary, the presented oral and intranasal mucosal vaccination strategies using *L. plantarum* offer tremendous potential for the development of new influenza vaccines.

## Supplementary Information

**Additional file 1: Figure S1. **Phylogenetic tree showing the genetic relationships of the six isolates from healthy pigs fecal samples with the closest sequences identified in GenBank by BLAST.

**Additional file 2: Figure S2.** HI and VN antibody titers in mice after immunization with the engineered *L. plantarum *strains.

**Additional file 3: Figure S3.** HI and VN antibody titers in mice after immunization with the engineered *L. plantarum *strains.

**Additional file 4: Table S1** Scoring criteria for selected probiotic *L. plantarum.*

## Data Availability

The authors will make available the raw data supporting the conclusions of this article upon reasonable request.

## Abbreviations

BSA: Bovine serum albumin
DMEM: Dulbecco’s modified Eagle medium
DPC: Days post-challenge
ELISA: Enzyme-linked immunosorbent assay
GIT: Gastrointestinal tract
GRAS: Generally recognized as safe
H&E: Haematoxylin and eosin
H1N1: Swine influenza A virus H1N1
HRP: Horseradish peroxidase
IIV: Inactivated influenza A virus
ILF: Intestinal lavage fluid
LB: Luria–Bertani
MRS: De Man, Rogosa and Sharpe
PBS: Phosphate-buffered saline
PCR: Polymerase chain reaction
SDS-PAGE: Sodium dodecyl sulphate–polyacrylamide gel electrophoresis
sIgA: Secretory IgA
swIAV: Swine influenza A virus
WIV: Whole inactivated virus
