# Supplementary figures and images for: Oral or intranasal immunization with recombinant Lactobacillus plantarum displaying head domain of Swine Influenza A virus hemagglutinin protects mice from H1N1 virus
Source: Microb Cell Fact. 2022 Sep 9;21:185. doi: 10.1186/s12934-022-01911-4 (PMC9461438; doi:10.1186/s12934-022-01911-4)

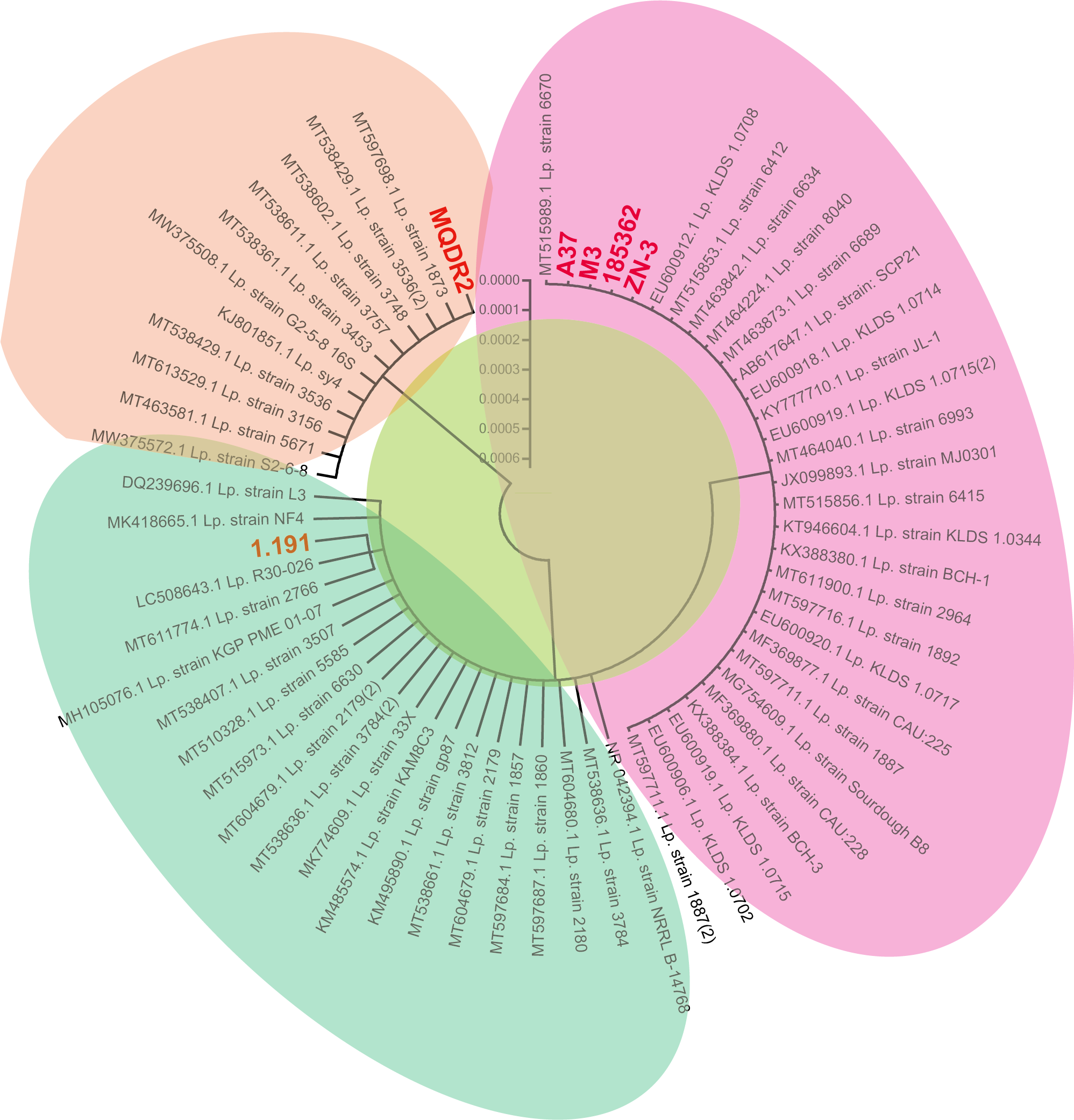

Supplement: Supplementary file 1 — Additional file 1: Figure S1. Phylogenetic tree showing the genetic relationships of the six isolates from healthy pigs fecal samples with the closest sequences identified in GenBank by BLAST. [file 12934_2022_1911_MOESM1_ESM.tif]

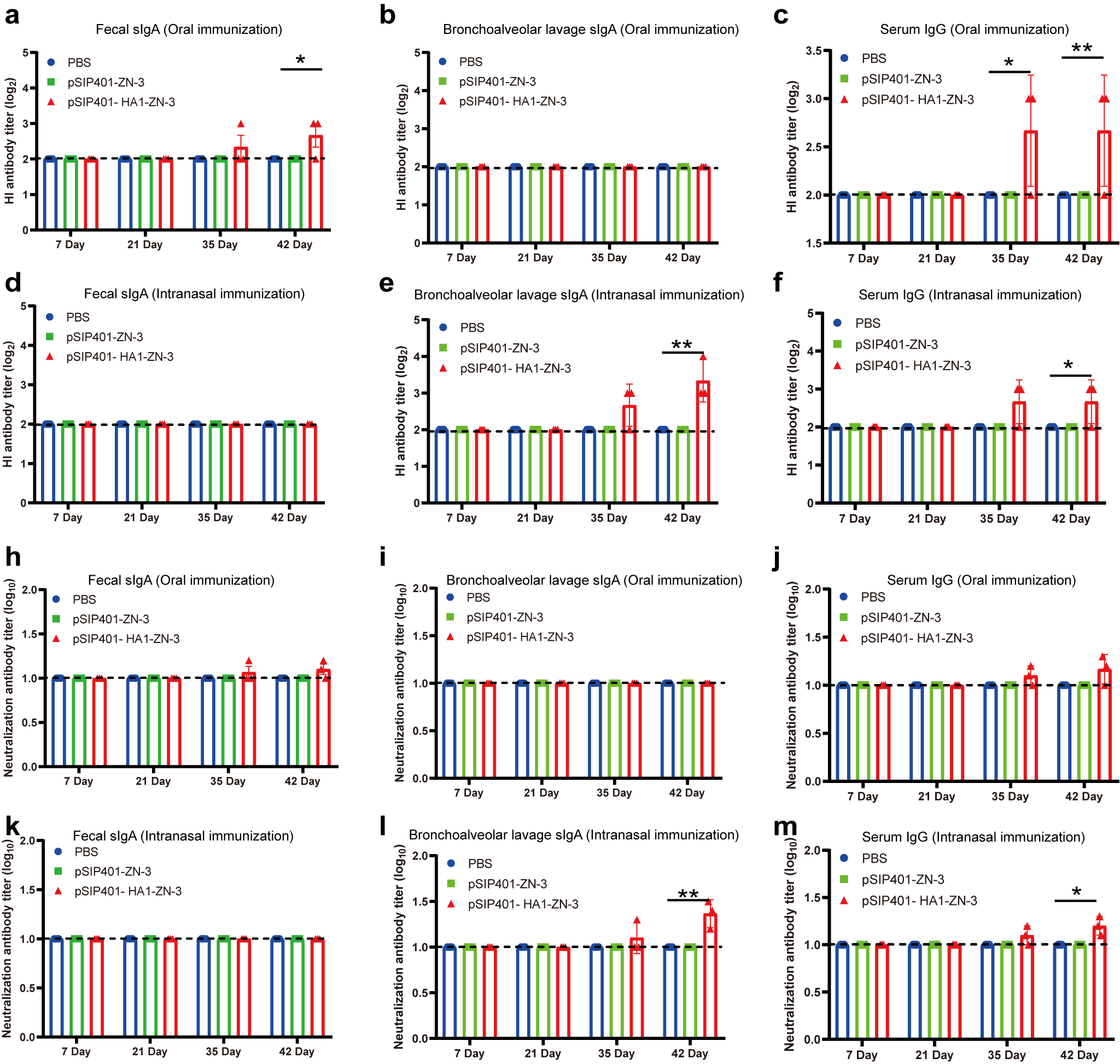

Supplement: Supplementary file 2 — Additional file 2: Figure S2. HI and VN antibody titers in mice after immunization with the engineered L. plantarum strains. [file 12934_2022_1911_MOESM2_ESM.tif]

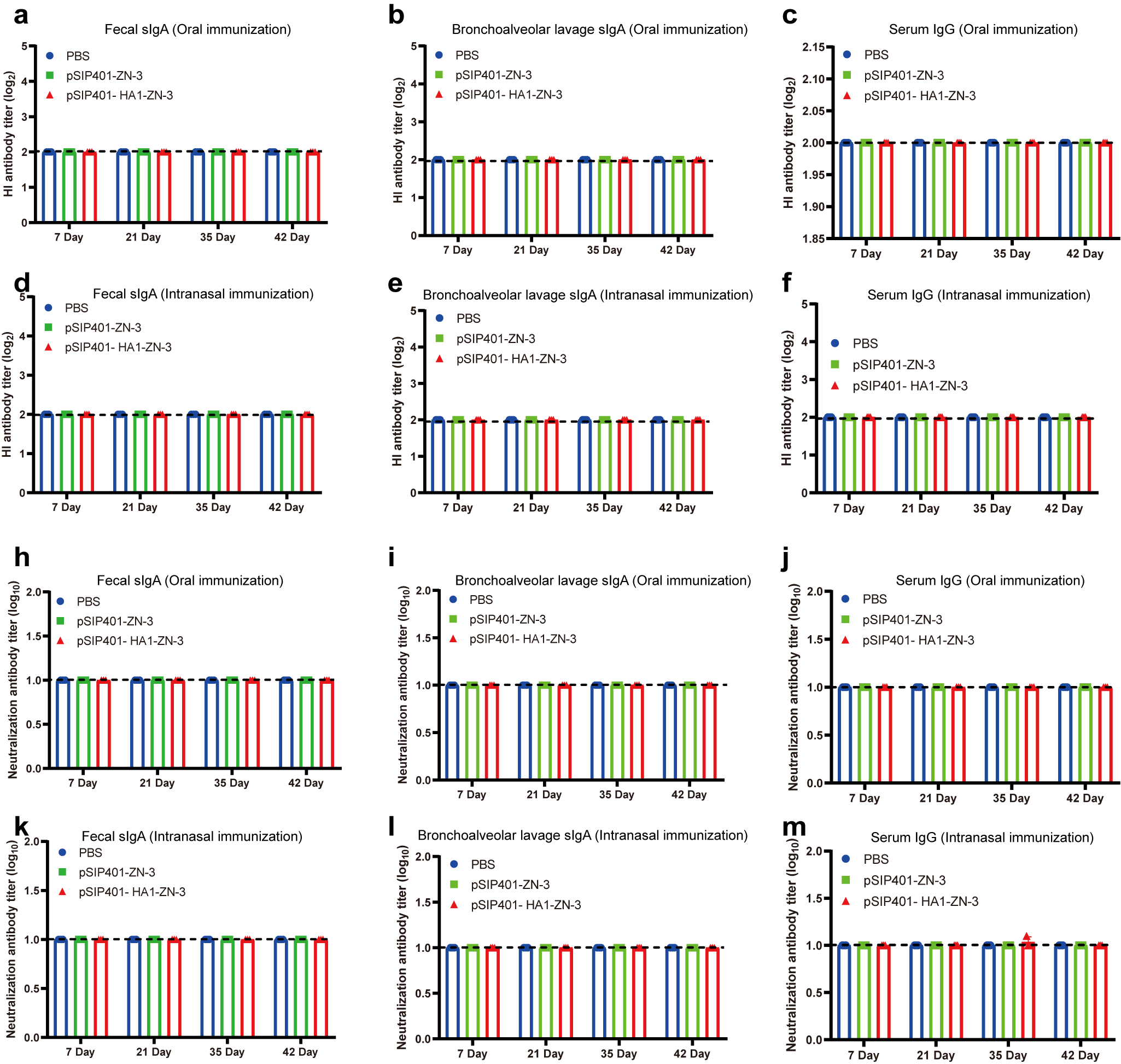

Supplement: Supplementary file 3 — Additional file 3: Figure S3. HI and VN antibody titers in mice after immunization with the engineered L. plantarum strains. [file 12934_2022_1911_MOESM3_ESM.tif]
