# Supplementary material for: Oral or intranasal immunization with recombinant Lactobacillus plantarum displaying head domain of Swine Influenza A virus hemagglutinin protects mice from H1N1 virus
Source: Microb Cell Fact. 2022 Sep 9;21:185. doi: 10.1186/s12934-022-01911-4 (PMC9461438; doi:10.1186/s12934-022-01911-4)
Supplement: Supplementary file 4 — Additional file 4: Table S1 Scoring criteria for selected probiotic L. plantarum. [file 12934_2022_1911_MOESM4_ESM.doc]

Additional file Table 1 Scoring criteria for selected probiotic *L. plantarum*

|  | **The expression level of exogenous proteins** | **Resistance to gastric juice** | **Resistance to bile salts** | **Antibiotic Susceptibility** | **Antibacterial activities** | **The adherence activity** | |
| --- | --- | --- | --- | --- | --- | --- | --- |
|  |  |  |  |  |  | **In vitro** | **In vivo** |
| **ZN-3** | ++++ | +++ | ++++ | ++++ | ++++ | ++++ | ++++ |
| **MQDR2** | ++++ | ++++ | ++ | ++ | ++++ | +++ | ++ |
| **1.191** | +++ | ++ | ++++ | +++ | ++++ | ++ | +++ |
| **185362** | ++ | - | - | - | - | - | - |
| **A37** | + | - | - | - | - | - | - |
| **M3** | + | - | - | - | - | - | - |

In this study, the expression level of exogenous proteins is classified as ++++, +++, ++, or +, which correspond to excellent, good, moderate, and poor states, respectively. Resistance to gastric juice and bile salts are same as above. Based on antibiotic susceptibility test *L. plantarum* strains were categorized into excellent (++++), good (+++), moderate (++), or poor (+). Antibacterial activities of Lactobacillus strains against gram-positive and gram-negative pathogens and the adherence activity are same as above.
